# MicroRNA-281-X Modulates Self-Grooming Behavior in Honeybees by Targeting Tyrosine Decarboxylase 2 in the Octopaminergic Pathway

**DOI:** 10.3390/insects17050522

**Published:** 2026-05-20

**Authors:** Yang Lü, Wenyao Ouyang, Jiali Liao, Liuchang Miao, Zhiguo Li, Songkun Su

**Affiliations:** 1 College of Bee Science, Fujian Agriculture and Forestry University, Fuzhou 350000, China; ly89617974@outlook.com (Y.L.);; 2 Heilongjiang Academy of Agricultural Sciences, Mudanjiang 157000, China

**Keywords:** self-grooming behavior, microRNA, tyrosine decarboxylase 2, octopamine, *Varroa destructor*, honeybees

## Abstract

Self-grooming is a fundamental and evolutionarily conserved behavior and is essential for removing *Varroa destructor* in honeybees (*Apis mellifera*). However, its molecular and neural regulation remains poorly understood. This study identifies a microRNA-mediated neuromodulatory pathway that governs the intensity of self-grooming behavior in *A. mellifera*. Comparative behavioral analyses revealed pronounced differences in grooming intensity between bees exhibiting strong (MS) and weak (MW) grooming phenotypes. Liquid chromatography detection and pharmacological experiments demonstrated that octopamine (OA) levels are significantly different between MS and MW bees, and OA enhances grooming behavior. Small RNA sequencing revealed differentially expressed miRNAs in the brains of MS versus MW bees, leading to the identification of miR-281-x as a candidate associated with OA regulation. Functional validation showed that overexpression of miR-281-x significantly reduces grooming behavior, whereas inhibition of miR-281-x enhances grooming. Mechanistically, bioinformatics prediction and experimental validation confirmed that miR-281-x directly targets the *tdc2*, which encodes tyrosine decarboxylase 2 in OA biosynthesis. Fluorescence in situ hybridization (FISH) revealed co-localization of miR-281-x and *tdc2* in Kenyon cells of the mushroom bodies. RNAi-mediated knockdown of *tdc2* significantly reduced grooming intensity and rescue experiments confirmed the miR-281-x–*tdc2*–OA regulatory axis. Together, our findings uncover a post-transcriptional modulatory mechanism linking a microRNA to neuromodulator-dependent behavioral plasticity, providing insight into the control of grooming intensity in honeybees.

## Simple Summary

Self-grooming behavior helps honeybees defend against *Varroa* mites and stay clean. We investigated how their brains control this behavior’s intensity and discovered a key modulator: microRNA-281-x acts as a molecular brake by limiting the production of octopamine, an important nerve signal. Artificially lowering this microRNA’s level increased octopamine and made bees groom more intensely. We located this system in specific brain regions, revealing a novel molecular pathway that tunes a vital behavior.

## 1. Introduction

The modulation of innate behavior in response to dynamic internal and environmental states is a hallmark of adaptive animal physiology. This behavioral plasticity is crucial for survival and fitness [1,2]. A central challenge in neuroscience is to understand how stereotyped motor programs are quantitatively tuned by molecular mechanisms within neural circuits [3,4]. Self-grooming represents a premier model behavior to address this question. It is an evolutionarily conserved, sequentially patterned action that serves essential functions across animal taxa, including maintenance of hygiene, thermoregulation, and parasite defense [5]. In insects, grooming effectively removes pathogens and ectoparasites [6,7,8,9]. The execution frequency and vigor of self-grooming, however, exhibit considerable individual variation [10,11,12,13], indicating that its underlying neural circuits are subject to modulatory influence rather than operating in a fixed manner. Uncovering the specific molecular pathways that confer this modulatory capacity is critical for advancing our understanding of behavioral control.

The honeybee (*A. mellifera*) offers a powerful and tractable model system for mechanistic studies of behavioral plasticity [14]. Its complex social organization is supported by a rich repertoire of behaviors that are modulated by well-defined neurochemical systems [15,16,17]. Among these, the biogenic amine octopamine (OA) serves as a master regulator of behavioral state in insects, functionally analogous to norepinephrine in vertebrates [18]. In honeybees, OA signaling orchestrates a diverse array of processes, including the regulation of appetite [19], division of labor [20], learning, and complex social communication such as the waggle dance [21,22]. OA has been pharmacologically and genetically implicated in the initiation and modulation of grooming behavior [13,23,24]. This positions the octopaminergic system as a central hub where various signals converge to adjust grooming activity [25], yet how OA synthesis is calibrated in specific neural contexts remains unclear. The biosynthesis of OA is catalyzed by the enzyme tyrosine decarboxylase encoded by the *tdc2* gene. Given that biosynthesis is the rate-limiting step in OA production, *tdc2* thus constitutes a principal regulatory node for neural circuit activity and behavior [26,27].

A growing body of evidence highlights the critical role of microRNAs (miRNAs) in regulating neuronal function and behavior [28,29,30]. As key post-transcriptional regulators, these endogenous small non-coding RNAs bind complementary sequences on target messenger RNAs, typically leading to transcript degradation or translational repression [31]. They are established modulators of neuronal development, excitability, and synaptic plasticity [32,33,34,35]. Moreover, miRNAs have been demonstrated to govern complex insect behaviors through the regulation of neurochemical pathways, from locomotor rhythms in *Drosophila* [36,37] to phase change in locusts [38,39] and division of labor in honeybees [40,41]. Nevertheless, a direct molecular link between a specific miRNA and the OA pathway has yet to be established. It also remains unknown whether such a link could explain natural variation in a defined behavior.

Here, we identify a functional miRNA-to-behavior pathway associated with self-grooming intensity in honeybees. We show that miR-281-x modulates OA synthesis by targeting *tdc2*. Using functional manipulations that both inhibit and enhance miR-281-x activity, we show that elevated miR-281-x suppresses grooming, while its inhibition enhances it. Bidirectional changes in miR-281-x levels produce predictable alterations in grooming vigor through corresponding effects on OA synthesis. Together, our findings define a miR-281-x/*tdc2*/OA axis that enables tuning of an innate behavior, providing a mechanistic framework for understanding how non-coding RNAs influence neuromodulatory control of behavioral plasticity.

## 2. Materials and Methods

### 2.1. Honeybees and Varroa Mites Collection

The honeybees (*A. mellifera ligustica*) used in this study were sourced from six genetically diverse, healthy colonies maintained at the experimental apiary of Fujian Agriculture and Forestry University, Fuzhou, China. These colonies were managed without the application of miticides or antibiotics for at least three months prior to the experiment to minimize the potential confounding effects of chemical treatments on bee physiology and behavior. To obtain age-matched cohorts for experimentation, frames of sealed brood were transferred from the source colonies to an incubator (34 ± 0.5 °C, 80 ± 5% relative humidity). Newly emerged adult bees were collected from these frames every 24 h. Each bee was gently collected and then individually marked on the dorsal thorax with using a non-toxic, odorless marker pen, and introduced into their original colony for natural rearing.

*Varroa* mites were collected using a modified version of the sugar roll method [42]. We collected mites using a plastic bucket with sugar powder and a fine sieve. Approximately 1000 bees were poured into the plastic bucket, avoiding the queen. Bees were rolled gently for 2–3 min until all the bees were well coated with powdered sugar. The bucket was inverted, and the sieve allowed mites to pass through while bees remained retained. Mites were transferred to Petri dishes with moist pieces of paper towel and placed in a laboratory room at 30 ± 0.5 °C.

### 2.2. Self-Grooming Behavior Assays

The intensity of self-grooming behavior was measured in the laboratory using the method described by Hamiduzzaman [43]. Briefly, a nine-day-old worker bee was individually placed into a Petri dish (90 mm × 15 mm) covered with a perforated lid. The bee was allowed to acclimatize to the new environment for 1 min. Following acclimatization, a *Varroa* mite was gently transferred onto the bee’s thorax using a fine paintbrush. The bee was then observed continuously for 3 min. All behavioral scoring was performed manually by a single trained observer.

The following two behavioral metrics were recorded for each bee:

First grooming time: The time interval (in seconds) between mite placement and the first detectable grooming action directed toward the mite.

Number of grooming bouts: A grooming bout was defined as a continuous, uninterrupted sequence of grooming actions (e.g., leg scraping, body shaking). A bout was considered terminated when the bee paused its grooming activity for more than 3 s.

Based on grooming efficacy and motor patterns, bees were classified into two discrete categories:

Strong groomers (MS): Bees that successfully dislodged the mite within the 3 min observation period. In addition, bees that did not achieve removal but exhibited vigorous, coordinated grooming involving three or more legs (rapid shaking or wiping motions) were also included in this category, consistent with the definition of “intense grooming” in previous studies [43].

Weak groomers (MW): Bees that failed to remove the mite and displayed limited grooming effort, characterized by slow, ineffective movements using no more than two legs. To confirm the stability of the weak groomer phenotype, a second mite-exposure trial was performed for all MW bees immediately following the first 3 min observation, using the same bee and a new mite. Only bees that showed weak grooming in both trials were retained as MW.

Bees that displayed no grooming behavior at all (i.e., no detectable grooming action during the entire 3 min observation) or showed inconsistent phenotypes between the first and second mite-exposure trials (e.g., weak grooming in the first trial but strong grooming in the second trial) were excluded from further analysis to ensure clear phenotype separation. Only bees with unequivocal MS or MW classification were used for subsequent molecular and pharmacological experiments.

Immediately after trials, each bee was flash-frozen in liquid nitrogen and stored at −80 °C for subsequent molecular analysis.

### 2.3. Small RNA Transcriptome Sequencing

The brains of nine-day-old MS and MW bees were collected and dissected. For each group, three independent biological replicates were prepared. Each replicate consisted of a pooled sample of 10 brains, resulting in a total sample size of 30 brains per replicate. Total RNA was extracted by Trizol reagent kit (Invitrogen, Carlsbad, CA, USA), and the RNA molecules in a size range of 18–30 nt were enriched by polyacrylamide gel electrophoresis (PAGE). Then, the 3′ adapters were added, and the 36–44 nt RNAs were enriched. The 5′ adapters were then ligated to the RNAs as well. The ligation products were reverse transcribed by PCR amplification, and the 140–160 bp size PCR products were enriched to generate a cDNA library and sequenced using Illumina NovaSeq X Plus by Gene Denovo Biotechnology Co. (Guangzhou, China). The small RNA libraries were deposited in the Sequence Read Archive database (accession number: PRJNA1227299). Differential expression analysis of miRNAs was performed by edgeR software (version 4.0) [44] between two different groups or samples. The Benjamini–Hochberg false discovery rate (FDR) method was applied to correct for multiple comparisons. miRNAs with |log_2_ fold change| ≥ 1 (fold change ≥ 2) and FDR < 0.05 were considered significantly differentially expressed between MS and MW groups.

### 2.4. Measurement of Neurotransmitter Levels in Honeybee Brains

Biogenic amine levels were measured using High-Performance Liquid Chromatography (HPLC; ACQUITY Arc, Waters, Milford, MA, USA) with Electrochemical Detection (ECD; 3465, Waters, USA). The extraction method for biogenic amine analysis was performed according to Huang [45]. Briefly, brains were dissected and homogenized in 200 µL of ice-cold buffer solution (mobile phase A). The samples were sonicated for 20 min in cold water, followed by centrifugation at 12,000× *g* for 20 min at 4 °C. The supernatants were filtered through 0.22 µm filters (Millipore, Burlington, MA, USA) into microvials for HPLC-ECD analysis.

Biogenic amines were isolated using an Acclaim^TM^ C18 reversed-phase column (2.2 μm, 2.1 × 100 mm) (Thermo Fisher Scientific, Waltham, MA, USA) at 40 °C. The autosampler was set at 10 °C. The mobile phase A consisted of 45 mM NaH_2_PO_4_, 1.7 mM sodium 1-octane sulfonate, and 50 mM 2Na-EDTA adjusted to pH 4.0 with citric acid, whereas phase B was acetonitrile. The mobile phase A is 90% and phase B is 10% equivalent elution. The flow rate was adjusted to 0.2 mL/min, and the injection volume was 10 µL. The total run time for each sample was 20 min mobile phase, during which detection was performed at 700 mV. OA, dopamine, tyramine, and 5-hydroxytryptamine (5-HT) levels were quantified using external standards (Figure S7). A standard curve was produced by serial diluting a standard solution containing OA, dopamine, tyramine, and 5-HT (Sigma-Aldrich, St. Louis, MO, USA).

### 2.5. Pharmacological Treatment of Honeybees

Given that OA effects on waggle dancing are dose-dependent and become significant only at 5 µg [21], and considering possible loss from topical application, we selected the same dose for OA treatment and we simultaneously confirmed the elevation of octopamine content in the bee brain following OA treatment (Figure S8). OA was dissolved in N,N′-dimethylformamide (DMF) at a concentration of 5 μg/μL, and control bees were treated with DMF. A total of 1 µL of the solution was topically applied gently on the thorax of the nine-day-old MS or MW bees [45]. Bees were held immobile for 30 s after treatment, to allow the solvent to penetrate the cuticle. After 30 min of the treatment, we performed the self-grooming assay to quantify the time of first grooming response and calculated the frequency of grooming of each bee. Due to sequential processing, observations were completed between 30–60 min post-treatment. Afterward, the honeybee samples were flash frozen using liquid nitrogen and stored at −80 °C for further analysis.

### 2.6. Assays of Quantitative PCR for miRNA and mRNA

Total RNA was extracted from tissues using the Eastep^®^ Super Total RNA Extraction Kit (Promega, Madison, WI, USA) according to the manufacturer’s instructions. Hiscript II Q RT SuperMix for qPCR (+gDNA wiper) and miRNA 1st Stand cDNA Synthesis Kit by stem-loop were used to prepare the primed cDNA and stem-loop cDNA, respectively (Vazyme, Nanjing, China). The miRNAs and mRNAs were subjected to qPCR using the miRNA Universal SYBR qPCR Master Mix and ChamQ Blue Universal SYBR qPCR Master Mix, respectively, according to the manufacturer’s instructions (Vazyme, Nanjing, China).

Biological replicates: Each biological replicate consisted of five bee brains pooled together as one sample. The exact number of biological replicates (n, i.e., number of independent pools) for each experimental group is indicated in the corresponding figure legend.

Technical replicates: Each biological sample (pooled brain RNA) was measured in three independent qPCR runs (technical triplicates). The mean Ct value of the three technical replicates was used for subsequent calculations.

Relative expression was calculated using the 2^−ΔΔCt^ method. β-actin and U6 were used as the internal controls of mRNA and miRNA, respectively. The qPCR primers are listed in Table S1.

### 2.7. miRNA Agomir and Antagomir Treatment In Vivo

The miRNA agomir or antagomir was chemically modified to overexpress and inhibit miRNAs in vivo, respectively [39,46,47]. Specifically, 60 pmol of agomir-281-x or antagomir-281-x (GenePharma, Shanghai, China) was injected into the brains of 15-day-old or six-day-old honeybees (anesthetized on the ice), respectively. Equivalent volumes of agomir-NC or antagomir-NC (GenePharma, Shanghai, China) also served as negative controls injected into the brains of honeybees. The treated honeybees were subjected to behavioral analysis after 72 h. Their brains were harvested, snap-frozen, and then stored at −80 °C.

### 2.8. Behavioral Rescue Experiments In Vivo

To rescue the phenotype, we administered the agomir/antagomir using the same in vivo delivery method as above described. In the first experiment, bees injected with agomir-281-x were topically treated on the dorsal thorax with 5 µg OA (in DMF) or DMF alone (control) 72 h later. After a 1 min permeation period, self-grooming behavior was recorded and analyzed between 30–60 min post-application. In the second experiment, bees injected with antagomir-281-x received a subsequent intracerebral injection of *sitdc2* or *siNC* (control) 24 h later. Self-grooming behavior was assessed 48 h after the final injection.

### 2.9. Protein Preparation and Western Blot Analysis

Honeybee brains were pooled (8 individuals per sample) to generate one biological replicate. Five independent biological replicates were analyzed per group for each of the following comparisons: (1) MS vs. MW, (2) agomir-NC vs. agomir-281-x, (3) antagomir-NC vs. antagomir-281-x. For each replicate, brains were homogenized in RIPA Lysis Buffer (Epizyme, Shanghai, China), and the total protein content of the lysates was determined using an Omni-Easy^TM^ Bicinchoninic Acid (BCA) protein assay kit (Epizyme, Shanghai, China). We separated the protein samples (20 µg) using gel electrophoresis and transferred them onto a membrane made of polyvinylidene fluoride (PVDF) (Millipore, USA), ready for antibody incubation. Membranes were blocked for non-specific binding using Protein Free Rapid Blocking Buffer (1×) (Epizyme, Cambridge, CA, USA) for 30 min, then incubated with the primary antibodies (rabbit anti-Tdc2 antibody, 1:500, Abcam; mouse anti-GAPDH antibody, 1:10,000, Proteintech, Wuhan, China) in Universal Antibody Dilution Buffer (Epizyme, Shanghai, China) overnight at 4 °C. After incubation, the membranes were washed, incubated with anti-rabbit IgG-HRP (MedChemExpress, Shanghai, China) or anti-mouse IgG-HRP (BOSTER, Wuhan, China) secondary antibody (1:20,000) for one hour at room temperature, and then washed again. Immunological detection was performed using Omni-ECL^TM^ Pico Light Chemiluminescence Kit (Epizyme, China). The intensity levels of the Western blot signals captured by the imaging system (Bio-Rad, Shanghai, China) were quantified using densitometry (Image J software, version 2.14.0). The relative expression of Tdc2 protein was calculated as the intensities of Tdc2 divided by the corresponding intensities of GAPDH.

### 2.10. Luciferase Report Assay

The ~500 bp coding sequence of 3′UTR surrounding the predicted miR-281-x target sites in *tdc2* was separately cloned into the pmirGLO vector (Promega, Madison, WI, USA) using the NheI and SacI restriction sites. To generate the mutation version, seven bp of binding sites corresponding to the seed region of the miRNA were mutated using Mut Express^®^ II Fast Mutagenesis Kit V2 (Vazyme, Nanjing, China) according to the manufacturer’s instructions to obtain the mut-*tdc2* construct. The HEK293T cells seeded on 24-well plates were co-transfected with 500 ng reporter plasmid and 20 uM miR-281-x mimic (Tsingke, Beijing, China) using Lipofectamine 3000 (Invitrogen, Waltham, MA, USA), and the mimic-NC (Tsingke, Beijing, China) was used as a negative control. The activities of the firefly and Renilla luciferases were measured after 24 h of transfection with the Dual-Glo Luciferase Assay System (Promega, Madison, WI, USA) using a Varioskan^TM^ LUX (Thermo Scientific, Waltham, MA, USA).

### 2.11. RNA Immunoprecipitation Assay (RIP)-qPCR Analysis

A Magna RIP kit (MilliporeSigma, Burlington, MA, USA) was used to perform a RIP assay according to the manufacturer’s instructions. The brains of honeybees were injected with a miR-281-x mimic, and mimic-NC was used as a negative control. Three independent biological replicates were performed. For each biological replicate, approximately 20 brains were collected 24 h post-injection, homogenized in ice-cold RIP lysis buffer, and stored at −80 °C overnight. A total of 5 µg of rabbit Ago-1 antibody (Abcam, Waltham, MA, USA) or normal rabbit IgG (MilliporeSigma, Burlington, MA, USA) was incubated with magnetic beads at room temperature. The frozen homogenates in the RIP lysates were thawed and centrifuged, 1/10 of the lysate was stored at −80 °C labeled as an “input” sample, and the supernatants were incubated with the magnetic bead–antibody complex at 4 °C overnight. Total RNA was extracted by TransZol Up (TransGen, Beijing, China) and reverse transcribed into cDNA using Hiscript II Q RT SuperMix for qPCR (+gDNA wiper) (Vazyme, Nanjing, China). qPCR was performed to quantify *tdc2*. The supernatants of the RIP lysates (input) and the IgG controls were assayed to normalize the relative expression levels of the target gene.

### 2.12. Co-Localization of miRNA and mRNA by Fluorescence In Situ Hybridization (FISH)

The in situ analysis of miR-281-x and *tdc2* in the bee brain was determined by the SweAMI-double FISH protocol (Servicebio, Wuhan, China). Briefly, digoxigenin-labeled miR-281-x and *tdc2* DNA probes were designed using miRBase and NCBI BLASTN (performed by Servicebio, China) and synthesized by Servicebio. After brain tissue paraffin blocks were sliced, samples were hybridized overnight at 40 °C with a diluted DNA probe (1:100). The washing process consists of washing the SSC solution (2×, 1×, 0.5×) with different layers of concentration for 5–10 min at 40 °C, respectively. After washing, add the corresponding branch probes as an intermediate hybridization medium for hybridization for 45 min at 40 °C. After washing, we added the corresponding signal probes (1:200) for three hours at 40 °C. After washing, tissue samples were stained with DAPI for 8 min and transferred to the mounting medium. The details of the DNA probes’ oligonucleotide sequences are listed in Table S2. Microscopy detection and collection of images using a laser confocal microscope (Zeiss, Oberkochen, Germany).

### 2.13. RNA Interference

The *dstdc2* was transcribed by a T7 RNAi Transcription Kit (Vazyme, Nanjing, China) according to the manufacturer’s instructions. We also synthesized *sitdc2* to knock down the *tdc2* (Table S1, Figure S6B). Specifically, each bee (anesthetized on the ice) was injected with 5 μg of *dstdc2*/*sitdc2* into the brain. Equivalent volumes of *dsEGFP*/*siNC* served as negative controls. The treated honeybees were subjected to behavioral analysis after 48 h. Their brains were harvested, snap-frozen, and then stored at −80 °C.

### 2.14. Statistical Analysis

GraphPad Prism version 8.0, SPSS 27.0 and R (4.5.3) software were used for statistical analysis. Normality and homogeneity of variance were assessed before analysis. Data are presented as means ± SEM for parametric analyses, or as median with interquartile range for nonparametric analyses. For comparisons between two independent groups with normally distributed data, Student’s *t*-test was used. Effect sizes were reported as Cohen’s d, and 95% confidence intervals (CIs) for the mean difference were calculated. This method was applied to qPCR validation of miRNA expression, RIP-qPCR, mRNA and protein levels, and octopamine concentration measurements. For comparisons between two independent groups with non-normally distributed data, the Mann–Whitney *U* test was used. Effect sizes were reported as Cliff’s Delta with 95% confidence intervals. This method was applied to behavioral assays. For paired comparisons (same individuals before and after treatment), the two-tailed Wilcoxon signed-rank test was used. Effect sizes were reported as r, along with 95% confidence intervals for the median difference. This method was applied to grooming behavior quantification following octopamine topical application. For comparisons involving more than two groups, the Kruskal–Wallis test was used, followed by Dunn’s post hoc test with Benjamini–Hochberg correction for multiple comparisons. Effect sizes were reported as eta-squared (η^2^) for tissue distribution analyses of miR-281-x and *tdc2*, or as r for pairwise comparisons in dual luciferase reporter assays. For neurotransmitter concentration comparisons among MS and MW groups, the same Kruskal–Wallis approach was applied. *p* < 0.05 was considered statistically significant. Significance levels are indicated as * *p* < 0.05, ** *p* < 0.01, *** *p* < 0.001; ns, not significant. For molecular and protein assays, the number of biological and technical replicates are specified in the corresponding Methods sections. For behavioral assays, sample sizes (n, number of bees per group) are provided in the respective figure legends.

## 3. Results

### 3.1. OA Influences Self-Grooming Behavior in A. mellifera

**Figure 1:**
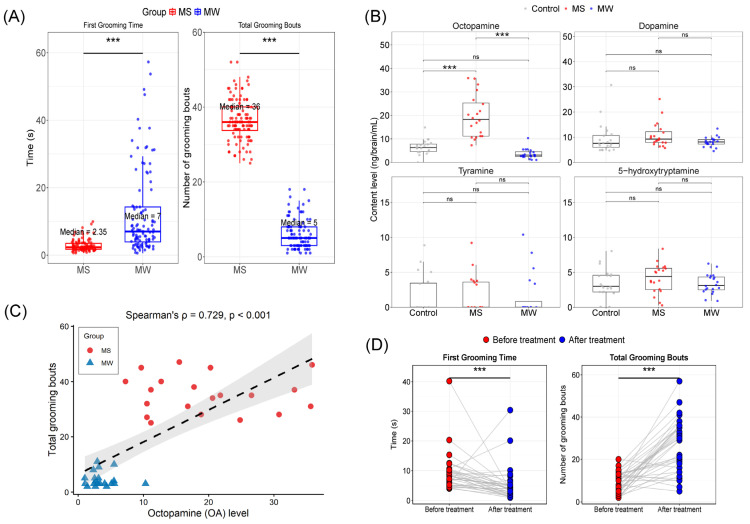
Neurotransmitters influence self-grooming behavior in *A. mellifera*. (**A**) Statistical comparison of the first grooming response time (s) and the total grooming bouts between MS (*n* = 108) and MW (*n* = 103) groups. Data were analyzed using the Mann–Whitney *U* test and are presented as median. Effect sizes are reported as Cliff’s Delta. (**B**) Concentrations of octopamine, dopamine, tyramine, and 5-HT in the brains of MS (*n* = 20) and MW (*n* = 20) bees (Kruskal–Wallis test (Dunn’s test with Benjamini–Hochberg correction), *** *p* < 0.001, ns *p* > 0.05). (**C**) Spearman correlation between brain octopamine levels and grooming bouts in MS and MW bees (*n* = 20 per group). The dashed line represents the linear regression trend line, and the gray shaded area around it indicates the 95% confidence interval. *ρ* and *p* values are indicated. (**D**) MW bees received a topical application of octopamine on the thorax. Time of the first grooming response and the total grooming bouts were quantified during 30–60 min periods (*n* = 36, Wilcoxon signed-rank test (paired, two-tailed), *** *p* < 0.001).

We observed individual differences in the grooming response of honeybees to *Varroa* mites. Bees exhibiting a strong grooming response were defined as strong groomers (MS), while those showing a weak response were defined as weak groomers (MW). From behavioral observations, 108 bees were classified as MS and 103 as MW. We recorded their first grooming time and total grooming bouts for these bees. The grooming phenotypes of MS and MW bees differed significantly in two key behavioral metrics (Figure 1A). Compared with MW bees, MS bees initiated the first grooming bout significantly sooner (median: 2.35 s vs. 7 s; Cliff’s Delta = −0.6944, *p* = 2.4 × 10^−11^, Mann–Whitney *U* test) and performed a significantly higher total number of grooming bouts during the observation period (median: 36 bouts vs. 5 bouts; Cliff’s Delta = 1; *p* = 2.0 × 10^−16^, Mann–Whitney *U* test).

To investigate whether neurotransmitters are involved in self-grooming behavior, we quantified the levels of four biogenic amines [16,48] in the brains of MS and MW bees using high-performance liquid chromatography (HPLC) with electrochemical detection (ECD). Honeybees that were not parasitized by *Varroa* mites were used as the control group for comparison. Our analysis revealed significantly elevated OA levels in the brains of MS bees compared to MW bees (Dunn’s test, *p* < 0.001, r = 0.832) and compared to control bees (*p* < 0.001, r = 0.511). No significant difference in OA levels was found between control and MW bees (*p* = 0.236, r = 0.321). The Kruskal–Wallis test confirmed an overall significant difference among the three groups (H = 42.27, df = 2, *p* < 0.001, η^2^ = 0.716). In contrast, no significant differences were observed between MS and MW bees for dopamine (*p* = 0.204 η^2^ = 0.0667) tyramine (*p* = 0.988 η^2^ = 0.0039) or 5-HT (*p* = 0.381 η^2^ = 0.0404) (Figure 1B).

To assess the relationship between OA levels and grooming behavior, we performed a Spearman correlation analysis between brain OA content and total grooming bouts in MS and MW bees. A significant positive correlation was observed (*ρ* = 0.729, *p* < 0.001, Figure 1C).

Furthermore, to test whether OA directly influences self-grooming behavior, we topically applied 5 μg of OA dissolved in DMF to the thorax of MW bees, with a control group receiving an equivalent volume of DMF alone. Following OA application, MW bees exhibited a significant reduction in the time to first grooming response (r = 0.643 *p* = 1.185 × 10^−4^) and a marked increase in total grooming frequency (r = 0.823 *p* = 1.238 × 10^−6^) (Figure 1C). Bees in the control group exhibited no changes in first response time and grooming frequency (Figure S1A,B).

These results demonstrate that OA is critical in modulating self-grooming behavior in *A. mellifera* and suggest that octopaminergic signaling may be an important neurochemical mechanism underlying grooming intensity.

### 3.2. The Brain miRNAs Profile Associated with Self-Grooming Behavior in A. mellifera

**Figure 2:**
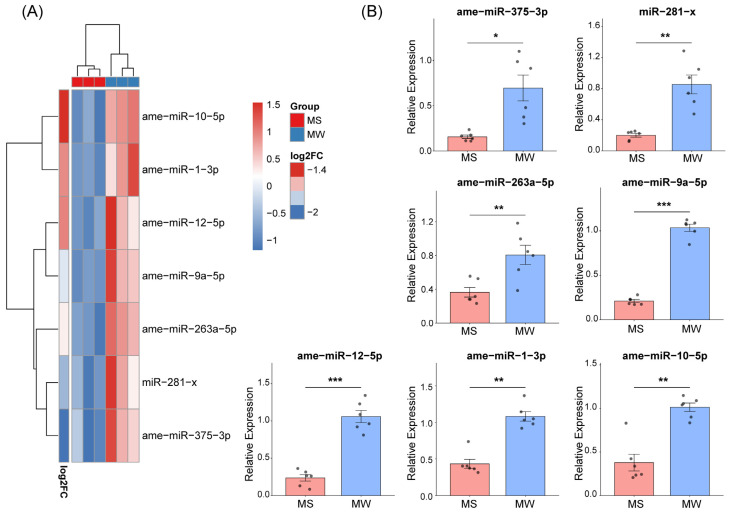
Identification of miRNAs potentially involved in self-grooming behavior in *A. mellifera*. (**A**) Top seven miRNAs expression profiles of MW and MS bees. (**B**) Validation of differentially expressed top seven miRNAs confirmed in the MW and MS bees by qPCR (*n* = 6 per group). Data are expressed as means ± SEM. The asterisks outside the strip indicate the significant difference between MW and MS groups by Student’s *t*-test, with effect sizes reported as Cohen’s d. * *p* < 0.05, ** *p* < 0.01, *** *p* < 0.001.

Given the established role of miRNAs in modulating neurotransmitter system [38,39], we performed small RNA sequencing on brain samples from MS and MW bees to investigate the molecular regulators underlying the differences in brain OA levels between the two groups. Differential expression analysis identified 11 miRNAs that were significantly different between MS and MW bees (FDR < 0.05, |log_2_FC| > 1). All 11 miRNAs showed higher expression in MW bees, whereas no miRNAs were upregulated in MS bees. To prioritize candidates, the top 7 miRNAs with the highest expression levels were selected. Among these, ame-miR-375-3p showed the largest fold change, followed by miR-281-x (Figure 2A).

We then validated the expression levels of these seven miRNAs using stem-loop qRT-PCR. The qRT-PCR results confirmed that all seven miRNAs were significantly upregulated in MW bees and downregulated in MS bees, consistent with the small RNA sequencing data. Specifically, compared to MS bees, MW bees exhibited significantly higher expression of ame-miR-375-3p (*p* = 1.25 × 10^−2^ Cohen’s d = 2.165), miR-281-x (*p* = 3.5 × 10^−3^ Cohen’s d = 3.07), ame-miR-263a-5p (*p* = 6.9 × 10^−3^ Cohen’s d = 2.008), ame-miR-9a-5p (*p* = 3.48 × 10^−8^ Cohen’s d = 10.595), ame-miR-12-5p (*p* = 1.61 × 10^−5^ Cohen’s d = 5.137), ame-miR-1-3p (*p* = 6.47 × 10^−5^ Cohen’s d = 4.183), and ame-miR-10-5p (*p* = 2.63 × 10^−4^ Cohen’s d = 3.409) (Figure 2B).

### 3.3. miR-281-x Is Associated with Self-Grooming Behavior in A. mellifera

**Figure 3:**
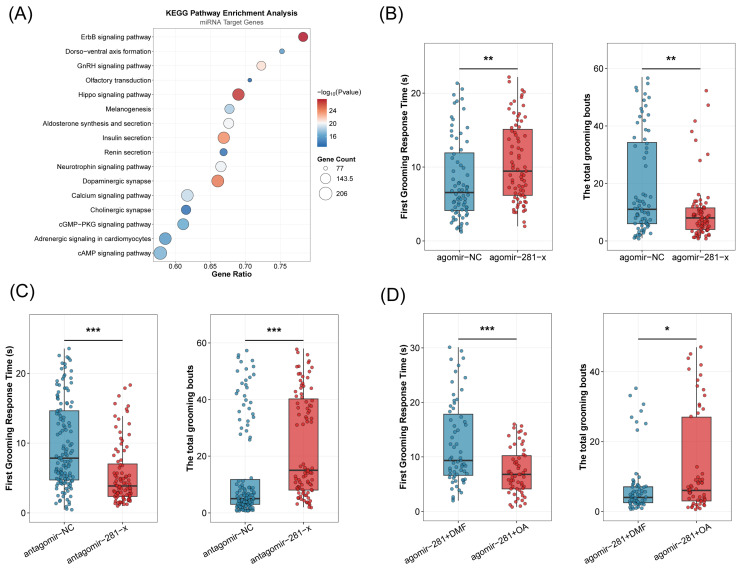
miR-281-x regulates the self-grooming behavior in *A. mellifera*. (**A**) Overview of KEGG pathways enriched for the target genes of seven miRNAs. (**B**,**C**) First grooming time (s) and total grooming bouts following agomir/antagomir-281-x injection. Group differences among agomir-NC (*n* = 72), agomir-281-x (*n* = 75), antagomir-NC (*n* = 130) and antagomir-281-x (*n* = 92) bees were tested by the Mann–Whitney *U* test. Effect sizes are reported as Cliff’s Delta. (**D**) Behavioral rescue assay in honeybees performed by topical application of octopamine (*n* = 103)/DMF (*n* = 78) to agomir-281-x-treated bees. First grooming time (s) and total grooming bouts were quantified. Statistical comparisons were performed using the Mann–Whitney *U* test, with effect sizes reported as Cliff’s Delta. * *p* < 0.05; ** *p* < 0.01, *** *p* < 0.001.

We next used miRanda and TargetScan to predict the potential target genes of the seven differentially expressed miRNAs. KEGG functional enrichment analysis revealed that these target genes were predominantly associated with pathways related to neural regulation and signal transduction (Figure 3A) Notably, among the predicted targets associated with OA biosynthesis, miR-281-x was identified as the exclusive miRNA targeting *tdc2* (encoding tyrosine decarboxylase 2), suggesting a potential regulatory role in self-grooming behavior.

Next, we experimentally investigated the functional role of miR-281-x in self-grooming behavior by manipulating its expression levels. Marked honeybees of the same emergence day were randomly assigned to treatment groups and injected with synthetic miRNA agomirs or antagomirs. The grooming behavior of injected bees was analyzed at 72 h post-injection and compared to that of negative control (NC) bees (Figure 3B). Quantitative analysis confirmed that miR-281-x expression was significantly elevated in the brains of bees injected with agomir-281-x (Figure S2A). Correspondingly, behavioral assays revealed that, compared to the agomir-NC group, the agomir-281-x group exhibited a significantly longer first grooming time (Cliff’s Delta = −0.2909, *p* = 2.35 × 10^−3^, Mann–Whitney *U* test) and a significantly lower total number of grooming bouts (Cliff’s Delta = 0.2928, *p* = 2.18 × 10^−3^, Mann–Whitney *U* test, Figure 3B). Conversely, miR-281-x expression was significantly reduced following antagomir-281-x injection (Figure S2B). Compared to the antagomir-NC group, the antagomir-281-x group showed a significantly shorter first grooming time (Cliff’s Delta = 0.4473, *p* = 1.4 × 10^−8^, Mann–Whitney *U* test) and a significantly higher total number of grooming bouts (Cliff’s Delta = −0.4773, *p* = 1.3 × 10^−9^, Mann–Whitney *U* test, Figure 3C). To assess whether the reduction in OA expression induced by miR-281-x overexpression underlies the suppression of grooming behavior, a rescue experiment was conducted. Application of 5 μg OA to the thorax of agomir-281-x-injected bees significantly restored grooming performance, as indicated by a significantly shorter first grooming time (Cliff’s Delta = 0.3957, *p* = 1.9 × 10^−4^, Mann–Whitney *U* test) and a significantly higher total number of grooming bouts compared to agomir-281-x alone (Cliff’s Delta = −0.2116, *p* = 4.53 × 10^−2^, Mann–Whitney *U* test, Figure 3D).

### 3.4. tdc2 Is a Direct Target of miR-281-x

**Figure 4:**
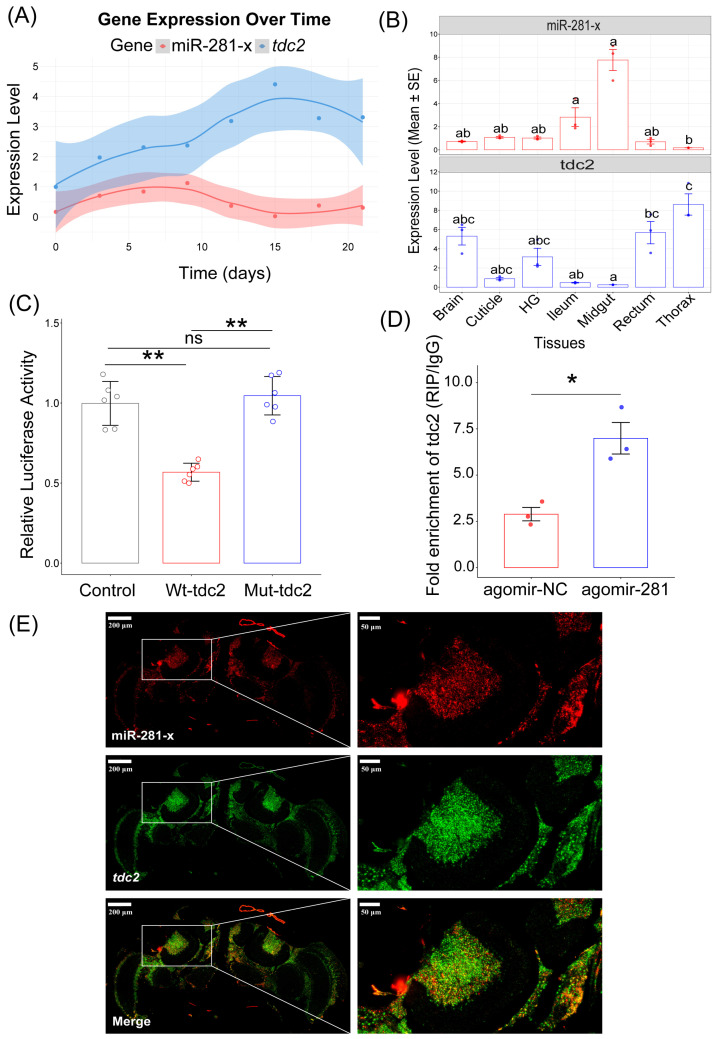
Tissue-specific expression profile and interactions between miR-281-x and its target *tdc2* in honeybees. (**A**) Relative expression levels of miR-281-x and *tdc2* from eclosion to 21-day-old adult bees. The smooth curve was fitted using the LOWESS spline method to visualize the dynamic expression of miR-281-x and *tdc2* (*n* = 3 per group). (**B**) Distribution of miR-281-x and *tdc2* gene expression in different tissues of honeybees (*n* = 3 per group, Kruskal–Wallis test). Different lowercase letters (a, b, c) represent significant differences (*p* < 0.05); ns denotes not significant (*p* ≥ 0.05). (**C**) Dual luciferase reporter assays in wild and mutant types of *tdc2* in vitro (*n* = 6 per group). Statistical comparisons were performed using the Kruskal–Wallis test, followed by post hoc pairwise comparisons. Effect sizes (r) for pairwise comparisons. ** *p* < 0.01. (**D**) RNA immunoprecipitation (RIP) was performed with an anti-Ago-1 antibody, and fold enrichment was quantified relative to rabbit IgG control. qPCR analysis was performed to amplify *tdc2* mRNA from the Ago-1 immunoprecipitates in brain tissue extracts treated with agomir-281-x compared with agomir-NC. Significant differences were determined by the Student’s *t*-test (*n* = 3 per group, * *p* < 0.05; ** *p* < 0.01). (**E**) miR-281-x and *tdc2* were co-labeled to determine the co-localization in the honeybee brain by fluorescence in situ hybridization (FISH). Where green (*tdc2*) and red signals (miR-281-x) overlap, a yellow signal is observed, indicating the co-localization of miR-281-x and *tdc2*. The images were visualized using a laser confocal microscope (Zeiss) at magnifications of ×12 (the left column) and ×40 (the right column), scale bars: 200 µm (left column) and 50 µm (right column).

To investigate interactions between miR-281-x and its target gene *tdc2*, we first analyzed the temporal expression profiles of both molecules in worker bee brains across developmental stages from newly emerged adults to 21 days post-eclosion. Our results demonstrated that miR-281-x expression increased from day 0 to day 9, after which it significantly decreased between days 9 and 15, stabilizing from days 18 to 21. In contrast, the expression of *tdc2* exhibited an inverse pattern, peaking during the later stages of the 21 days (Figure 4A). We also evaluated the tissue distribution of miR-281-x and *tdc2* in bees of the same age. Both miR-281-x and *tdc2* were expressed in all seven tissues analyzed, though they exhibited distinct expression patterns (Figure 4B).

To directly test whether miR-281-x can degrade *tdc2* or inhibit its translation, we performed reporter assays using dual-luciferase constructs that were fused to the 3′ untranslated region (UTR) of *tdc2*. These assays confirmed that miR-281-x directly binds to *tdc2*, as evidenced by decreased luciferase activity in the presence of the miR-281-x mimic and *tdc2* binding fragments (Kruskal–Wallis test, *p* = 0.005, r = 0.832). In contrast, luciferase activity remained unchanged when the *tdc2*’s 3′ UTR contained mutant binding sequences (Kruskal–Wallis test, *p* = 0.689, r = 0.139) (Figure 4C). To further validate this interaction in vivo, we performed RNA immunoprecipitation (RIP) assays using an AGO1 antibody [49], which is a key protein involved in miRNA-mediated gene silencing. RIP results revealed that brains treated with the agomir-281-x exhibited significantly enriched levels of *tdc2* compared to those treated with the miR-281-x negative control (*p* = 1.15 × 10^−2^ Cohen’s d = 3.612, Figure 4D), further supporting the association of miR-281-x and *tdc2* transcripts within the AGO1 complex in vivo. Finally, we examined the cellular localization of miR-281-x and *tdc2* in the honeybee brain using double-labeling fluorescence in situ hybridization (FISH). Our results demonstrated the co-localization of miR-281-x and *tdc2* in both the KCs of the MB and the optic lobes. Notably, these regions are known to be involved in complex behaviors, including the integration of sensory, olfactory, and visual information [50,51] (Figure 4E). As a negative control, no signals were detected in the same brain regions when labeled with the corresponding control probes (Figure S3). Collectively, these findings demonstrate that miR-281-x directly binds to the *tdc2* 3′UTR and associates with the AGO1-containing silencing complex in whole bee brain tissue. The co-localization of both molecules in mushroom body neurons (Kenyon cells) and optic lobes supports the potential for a functional interaction in neurons, although direct evidence of miR-281-x-mediated inhibition of *tdc2* specifically in honeybee neurons remains to be established.

### 3.5. miR-281-x Influences OA Signaling by Targeting tdc2 and Modulates Self-Grooming Behavior in A. mellifera

**Figure 5:**
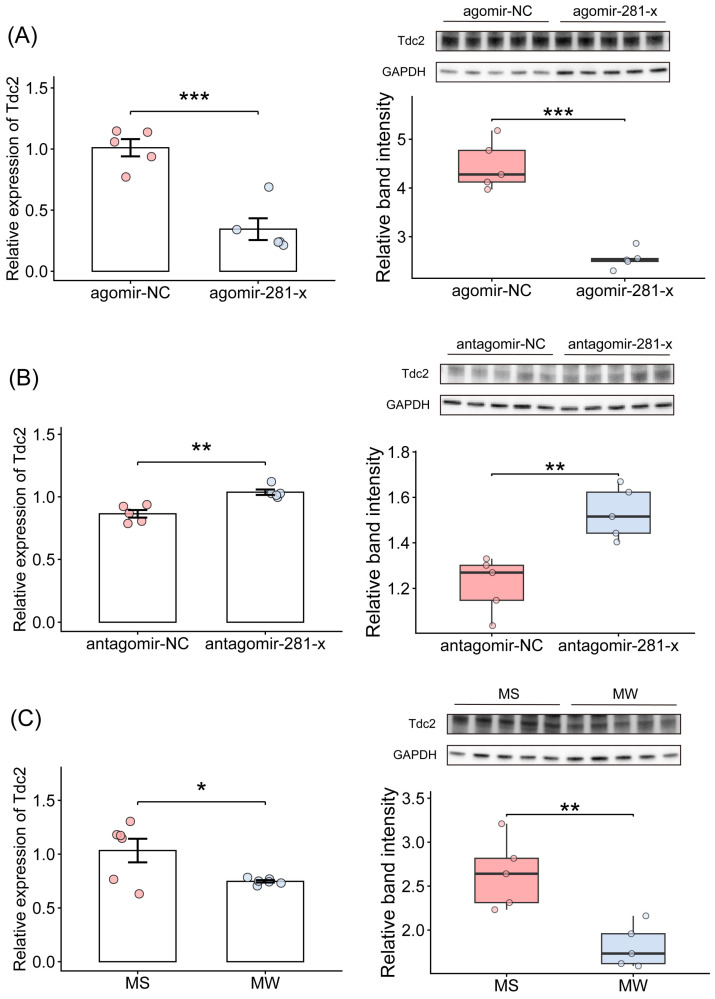
miR-281-x negatively regulates the expression of *tdc2*. (**A**) Relative mRNA level (*n* = 5 per group) and protein level (*n* = 5 per group) of *tdc2* in the brains of bees injected with agomir-281-x. (**B**) Relative mRNA level (*n* = 5 per group) and protein level (*n* = 5 per group) of *tdc2* in the brains of bees injected with antagomir-281-x. (**C**) Relative mRNA level (*n* = 6 per group) and protein level (*n* = 5 per group) of *tdc2* in the brains of MS and MW bees. The data are presented as the mean ± SEM; The asterisks outside the strip indicate the significant difference between controls and the treatments by Student’s *t*-test. * *p* < 0.05, ** *p* < 0.01, *** *p* < 0.001. Protein: Tdc2: 72 kDa; GAPDH: 36 kDa.

Given that miR-281-x directly interacted with *tdc2*, we determined the mRNA and protein expression levels of *tdc2* using miR-281-x overexpression and knockdown experiments in vivo. The mRNA and protein expression levels of *tdc2* were down-regulated by injecting agomir-281-x as compared with the control group injected with agomir-NC (mRNA: *p* = 3.72 × 10^−4^ Cohen’s d = 3.716, protein: *p* = 4.55 × 10^−5^ Cohen’s d = 5.031) (Figure 5A and Figure S4A,B). By contrast, the mRNA and protein levels of *tdc2* increased in the brains of honeybees injected with antagomir-281-x compared with those treated with antagomir-NC (mRNA: *p* = 1.67 × 10^−3^ Cohen’s d = 2.932, protein: *p* = 3.0 × 10^−3^ Cohen’s d = 2.655) (Figure 5B and Figure S4C,D). Next, to examine whether *tdc2* expression correlates with the intensity of self-grooming behavior, we compared the mRNA and protein levels of *tdc2* in the brains of MS and MW bees. *tdc2* expression was significantly higher in MS bees, which exhibit stronger grooming behavior, compared to MW bees (mRNA: *p* = 4.75 × 10^−2^ Cohen’s d = 1.499, protein: *p* = 4.01 × 10^−3^ Cohen’s d = 2.523) (Figure 5C and Figure S5).

**Figure 6:**
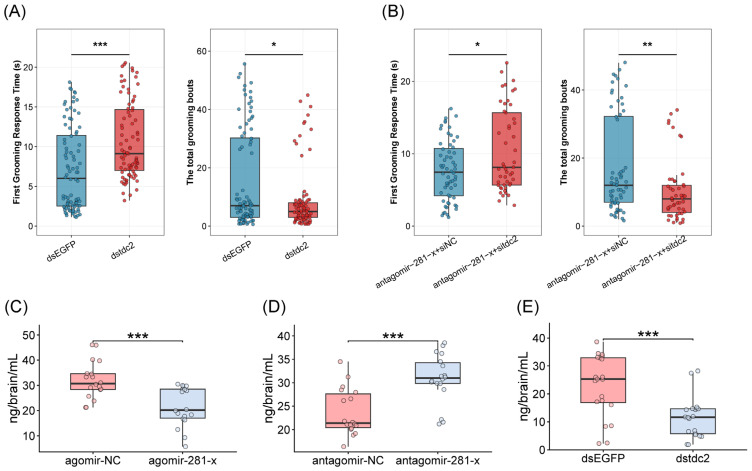
miR-281-x controls octopamine signaling by targeting *tdc2* modulates self-grooming behavior. (**A**) First grooming time (s) and total grooming bouts after injecting *dstdc2*. Group differences among *dsEGFP* (*n* = 88) and *dstdc2* (*n* = 83) bees were tested by the Mann–Whitney *U* test, ** *p* < 0.01. (**B**) Behavioral rescue assay was performed by injecting *sitdc2* into honeybees pretreated with antagomir-281-x (the Mann–Whitney *U* test * *p* < 0.05, antagomir-281-x + *siNC*: *n* = 60; antagomir-281-x + *sitdc2*: *n* = 49). (**C**,**D**) Concentrations of octopamine in the brains of bees (*n* = 18 per group) injected with agomir-281-x and antagomir-281-x. (**E**) Concentrations of octopamine in the brains of bees (*n* = 20 per group) with *tdc2* RNAi knockdown. The data are presented as the mean ± SEM; The asterisks outside the strip indicate the significant difference between controls and the treatments by Student’s *t*-test. * *p* < 0.05, ** *p* < 0.01, *** *p* < 0.001.

To further explore the functional role of *tdc2* in regulating self-grooming behavior, we performed a knockdown experiment by injecting *tdc2*-specific dsRNA into the brains of worker bees. Seventy-two hours after injection, we observed a significant reduction in the mRNA expression (Figure S6A). Behavioral assays revealed that, compared to the *dsEGFP* control group, the *dstdc2* group exhibited a significantly longer first grooming time (Cliff’s Delta = −0.407, *p* = 4.37 × 10^−6^, Mann–Whitney *U* test) and a significantly lower total number of grooming bouts (Cliff’s Delta = 0.201, *p* = 2.30 × 10^−2^, Mann–Whitney *U* test) (Figure 6A). Meanwhile, we conducted a complementary rescue assay by silencing *tdc2* expression to counteract the increased grooming induced by antagomir-281-x. The results showed that, compared with the *siNC* control group, bees injected with *sitdc2* exhibited a significantly shorter first grooming time (Cliff’s Delta = −0.2585, *p* = 2.07 × 10^−2^, Mann–Whitney *U* test) and a significantly higher total number of grooming bouts (Cliff’s Delta = 0.3422, *p* = 2.18 × 10^−3^, Mann–Whitney *U* test) (Figure 6B).

As *tdc2* is an essential gene involved in OA signaling, which is crucial for a range of behaviors, including self-grooming, we next sought to determine whether miR-281-x also regulates OA levels. We measured the OA levels of the worker bees injected with agomir-281-x or antagomir-281-x by using HPLC with ECD. The results showed that OA contents in the brains of worker bees were significantly reduced by injecting agomir-281-x compared with the group injected with agomir-NC (*p* = 2.0 × 10^−4^ Cohen’s d = 1.389, Figure 6C). Meanwhile, the OA contents in the brains of worker bees were increased dramatically by injecting antagomir-281-x compared with the injected antagomir-NC (*p* = 1.49 × 10^−4^ Cohen’s d = 1.423, Figure 6D). Additionally, OA levels were significantly reduced in the brains of *tdc2*-knockdown bees compared to the control group injected with *dsEGFP* (*p* = 3.97 × 10^−4^ Cohen’s d = 1.228, Figure 6E). The change in OA levels in the brains of worker bees was consistent with *tdc2* expression by using miR-281-x overexpression and knockdown; therefore, miR-281-x influences OA signaling by negatively regulating the expression of *tdc2*.

## 4. Discussion

**Figure 7:**
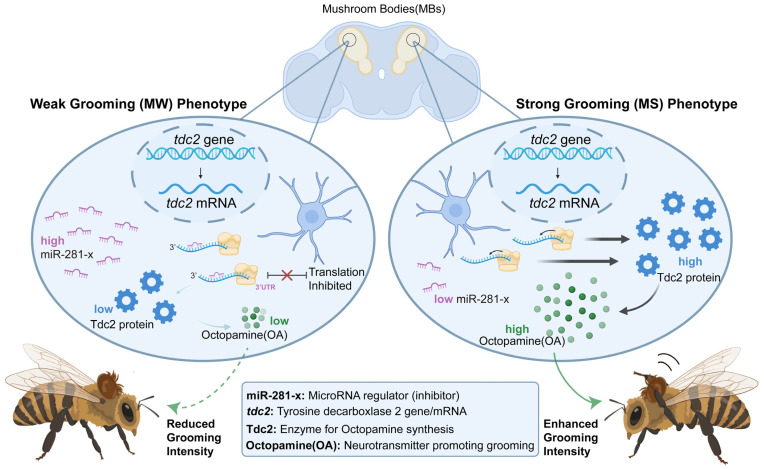
The miR-281-x–*tdc2*–octopamine axis regulates self-grooming in *A. mellifera*. miR-281-x acts as a negative regulator by directly targeting *tdc2*, a key enzyme for octopamine synthesis. High miR-281-x levels inhibit *tdc2* translation and reduce octopamine, resulting in weak grooming. Conversely, low miR-281-x levels facilitate *tdc2* expression and octopamine accumulation, driving intense self-grooming behavior.

This study elucidates a post-transcriptional regulatory pathway that quantitatively modulates an innate defensive behavior in honeybees. We identify a regulatory axis linking a specific brain-enriched miR-281-x to differential self-grooming intensity through its targeted regulation of OA biosynthesis (Figure 7).

Our initial miRNA profiling revealed a pronounced asymmetry in miRNA expression between MS and MW bees, with most differentially expressed miRNAs upregulated in weak groomers. This expression pattern raises the hypothesis that some of these miRNAs may negatively modulate grooming-related neural pathways, a possibility that is consistent with previously established roles of miRNAs as negative modulators of neuronal excitability and behavioral output in insects [52,53,54]. However, direct functional tests would be required to determine whether this asymmetric expression translates into actual inhibitory regulation of grooming behavior. In addition, the target genes of candidate miRNAs associated with honeybee grooming behavior are significantly enriched in a coordinated network comprising olfactory perception, neurotransmitter regulation, and cellular signaling hubs. This observation suggests a potential link between these miRNAs and multiple biological processes relevant to grooming, including the integration of external olfactory stimuli, neural signals for behavioral decision-making, and physiological homeostasis regulation [55,56,57]. Taken together, these analyses support a hypothesis in which the regulatory network associated with the identified miRNAs connects sensory input, motor coordination, and energy balance. While these findings provide new insights into candidate molecular mechanisms underlying social insect behavioral regulation, functional validation remains necessary to establish causal relationships.

We found that miR-281-x is upregulated in MW bees. These bees also remove mites less effectively, which suggests a functional link between miR-281-x and grooming behavior. Previous work has shown that miR-281-x in the honeybee brain is not just a developmental regulator, it is also highly responsive to pesticides and has been linked to neural damage and behavioral changes [58,59]. If high expression of miR-281-x reflects a dysfunction in neural circuits, or directly causes such dysfunction, then it would explain why these bees show reduced grooming behavior. At the same time, miR-281-x in bees is regulated by juvenile hormone and 20-hydroxyecdysone and suppresses *EcR* and *ftz-f1* during pupal metamorphosis [60], and these hormone pathways do not disappear in adults, they still influence learning, memory and other behaviors [61,62,63]. Thus, the upregulation of miR-281-x in MW bees can be explained by two possible mechanisms. One possibility is that abnormal hormone signaling during development left a permanent mark on brain wiring. Another is that ongoing regulation in the adult directly suppresses grooming through *EcR*-related pathways. Either way, our study suggests that miR-281-x is not just a marker of development or stress, it connects hormone state, neural function and social immunity. Whether these proposed mechanisms hold true awaits future experimental validation.

Our results confirm a positive correlation between OA levels and grooming intensity in bees. This aligns with the established role of OA in regulating insect grooming [13,23,24] that suggests that OA serves as a key quantitative signal driving this behavior. What our study adds is an understanding of how OA levels are influenced. We found that miR-281-x directly targets *tdc2*, the rate-limiting enzyme in OA biosynthesis. This identifies miR-281-x as an upstream modulator that helps set OA levels, influencing this signaling pathway. The concept of a miRNA controlling a neurotransmitter is not without precedent. In mice, miR-218 controls dopamine release by regulating the intrinsic excitability of dopaminergic neurons [35]. More directly relevant to our findings, miR-669g targets TPH2, the rate-limiting enzyme for 5-HT synthesis, and its overexpression reduces brain 5-HT levels and induces behavioral deficits [64]. Similarly, miR-326 and miR-9 regulate dopamine D2 receptor expression, thereby modulating dopaminergic signaling [65]. Together these examples and our findings suggest that microRNAs regulate neurotransmitter systems by targeting biosynthetic enzymes or receptors.

Notably, we observed no significant difference in tyramine (the direct precursor of OA [66]) levels between MS and MW groups. This indicates that the behavioral divergence likely depends not on the static accumulation of tyramine, but rather on its rate of conversion to OA under pressure [66,67]. Our study did not directly test this conversion step. But one explanation is that the activity of tyramine β-hydroxylase (the enzyme catalyzing this final reaction) might be regulated in response to the mite challenge, thereby controlling the OA production scaling the grooming output [68]. This hypothesis will require direct measurement of enzyme activity or flux analysis in future studies.

Our tissue expression analysis showed that *tdc2* is expressed at the highest level in honeybee thoracic muscle. This distribution pattern is consistent with the biological function of OA. Octopaminergic neurons directly project onto flight muscle fibers, the octopamine β receptor is highly expressed in flight muscle, and octopamine signaling is required for honeybee thermogenesis [69,70]. This indicates that OA needs to be synthesized locally at the site of motor execution, and the high expression of *tdc2* in the thorax meets this demand. This finding has important implications for understanding grooming behavior. Grooming depends on motor output driven by the thorax. Bees use their middle and hind legs to scrape their body and these movements are controlled by the thoracic ganglion. OA stimulates neuromuscular transmission and locomotion, and bees infected with certain viruses show increased expression of octopamine receptors along with enhanced flight capacity [69]. Thus, the high expression of *tdc2* in the thorax is not accidental but reflects the local demand for OA in motor regulation. But this raises the question of how inhibition of *tdc2* by miR-281-x in the brain affects thoracic grooming movements. Studies have shown that descending octopaminergic neurons exist in the insect brain, which project directly to the thoracic ganglion and regulate motor neuron excitability [71]. In honeybees, OA-like immunoreactive neurons are widely distributed in the brain and extend downward to the thoracic ganglion [72]. We hypothesize that inhibition of *tdc2* by miR-281-x in the brain may reduce the excitability of thoracic motor neurons by weakening the output of these descending octopaminergic neurons. Testing this hypothesized descending pathway will be an important goal for future studies.

Several limitations of this study should be acknowledged. Our work reveals a molecular pathway that regulates self-grooming behavior in honeybees, a key defensive response against *Varroa destructor*. However, we did not directly test whether this regulatory mechanism helps bees resist mite infestation under natural conditions. Future field studies are needed to examine the functional relevance of this pathway in realistic colony settings. Another limitation is our focus on miR-281-x as a prioritized candidate rather than systematically evaluating all differentially expressed miRNAs. Other differentially expressed miRNAs identified in our profiling may also influence grooming behavior either independently or in coordination with miR-281-x. Future mechanistic studies should characterize the roles of these additional miRNAs. In addition, octopamine affects a wide range of behaviors in honeybees beyond grooming. Whether the miR-281-x–*tdc2* regulatory axis also modulates other octopamine-dependent processes remains to be determined. We acknowledge that some of the mechanisms discussed above—including descending octopaminergic pathways and endocrine regulation by juvenile hormone and 20-hydroxyecdysone—remain hypothetical and await experimental testing. Addressing these questions will provide a more complete understanding of how miRNA-mediated regulation shapes behavioral plasticity in social insects.

## 5. Conclusions

In conclusion, this study uncovers a post-transcriptional modulatory mechanism that influences self-grooming intensity in honeybees. We demonstrate that miR-281-x directly targets *tdc2* for OA biosynthesis. The miR-281-x–*tdc2*–OA axis influences whether a bee grooms strongly or weakly. Our findings reveal how the miRNA can shape behavioral plasticity by controlling the synthesis of a key neuromodulator. This offers a molecular entry point into understanding individual variation in social immunity and innate defensive behaviors in insects.

## Data Availability

The original contributions presented in this study are included in the article/Supplementary Materials. Further inquiries can be directed to the corresponding authors.

## Supplementary Materials

The following supporting information can be downloaded at: https://www.mdpi.com/article/10.3390/insects17050522/s1, Figure S1: Neurotransmitters influence self-grooming behavior in *A. mellifera*. (A,B) MW bees received a topical application of DMF on the thorax as the control group. Time of the first grooming response and the total grooming bouts were quantified during 30–60 min periods (*n* = 20 per group, Wilcoxon signed-rank test); Figure S2: The expression level of miR-281-x of honeybees. (A,B) The miR-281-x relative expression was injected with agomir-281-x and antagomir-281-x (*n* = per group). The data are presented as the mean ± SEM. The asterisks outside the strip indicate the significant difference between controls and the treatments by Student’s *t*-test. *** *p* < 0.001; Figure S3: The negative control for co-localization of miR-281-x and *tdc2* in the honeybee brain. The images were visualized using a confocal fluorescence microscope (Zeiss), Scran bar 400 µm; Figure S4: The western blot scans of Tdc2. (A,C) The western blot scans of Tdc2 in the honeybee brains injected with agomir-281-x and antagomir-281-x (*n* = 5 per group). (B,D) There were original images of agomir-281-x and antagomir-281-x, respectively; Figure S5: The western blot scans of Tdc2. (A,B) The western blot scan of Tdc2 in the MS and MW bee brains and the original image (*n* = 5 per group); Figure S6: The expression level of *tdc2* of honeybees. (A, B) The *tdc2* relative expression was injected with *dsEGFP* and *dstdc2* or *siNC* and *sitdc2* (*n* = 6 per group). The data are presented as the mean ± SEM; The asterisks outside the strip indicate the significant difference between controls and the treatments by Student’s *t*-test. *** *p* < 0.001; Figure S7: HPLC-ECD chromatogram of biogenic amines. Depicts a representative calibration curve using octopamine, dopamine, tyramine, and serotonin standards ranging from 5 ng to 0.5 µg. Red line: 5 ng/mL; green line: 10 ng/mL; black line: 20 ng/mL; purple line: 50 ng/mL; pink line: 100 ng/mL; blue line: 200 ng/mL; cyan line: 500 ng/mL; Figure S8: Octopamine levels in honeybee brain 30 min after topical application of DMF or 5 µg OA. Data are shown as mean ± SEM. ** *p* < 0.01, unpaired two-tailed Student’s *t*-test (*n* = 3 per group); Table S1: Primers used in this study; Table S2: Probe sequence used in this study.

## References

[1] Xiao W., Jiao Z.L., Senol E., Yao J., Zhao M., Zhao Z.D., Chen X., Cao P., Fu Y., Gao Z. (2022). Neural circuit control of innate behaviors. Sci. China Life Sci..

[2] Su C.Y., Wang J.W. (2014). Modulation of neural circuits: How stimulus context shapes innate behavior in *Drosophila*. Curr. Opin. Neurobiol..

[3] Wei D., Talwar V., Lin D. (2021). Neural circuits of social behaviors: Innate yet flexible. Neuron.

[4] Devineni A.V., Scaplen K.M. (2021). Neural Circuits Underlying Behavioral Flexibility: Insights from *Drosophila*. Front. Behav. Neurosci..

[5] Kalueff A.V., Stewart A.M., Song C., Berridge K.C., Graybiel A.M., Fentress J.C. (2016). Neurobiology of rodent self-grooming and its value for translational neuroscience. Nat. Rev. Neurosci..

[6] Mehmood N., Hassan A., Zhou W., Usman H.M., Ai H., Huang Q. (2022). Behavioural alterations in female *Aedes aegypti* mosquito in response to entomopathogenic fungal infections. Pest Manag. Sci..

[7] Reber A., Purcell J., Buechel S.D., Buri P., Chapuisat M. (2011). The expression and impact of antifungal grooming in ants. J. Evol. Biol..

[8] Zhukovskaya M., Yanagawa A., Forschler B.T. (2013). Grooming Behavior as a Mechanism of Insect Disease Defense. Insects.

[9] Le Conte Y., Meixner M.D., Brandt A., Carreck N.L., Costa C., Mondet F., Büchler R. (2020). Geographical Distribution and Selection of European Honey Bees Resistant to *Varroa destructor*. Insects.

[10] Arechavaleta-Velasco M.E., Alcala-Escamilla K., Robles-Rios C., Tsuruda J.M., Hunt G.J. (2012). Fine-scale linkage mapping reveals a small set of candidate genes influencing honey bee grooming behavior in response to *Varroa* mites. PLoS ONE.

[11] Mustard J.A., Pham P.M., Smith B.H. (2010). Modulation of motor behavior by dopamine and the D1-like dopamine receptor AmDOP2 in the honey bee. J. Insect. Physiol..

[12] Blenau W., Baumann A., Farooqui T., Farooqui A.A. (2016). Chapter 14—Octopaminergic and Tyraminergic Signaling in the Honeybee (*Apis mellifera*) Brain: Behavioral, Pharmacological, and Molecular Aspects. Trace Amines and Neurological Disorders.

[13] Fussnecker B.L., Smith B.H., Mustard J.A. (2006). Octopamine and tyramine influence the behavioral profile of locomotor activity in the honey bee (*Apis mellifera*). J. Insect Physiol..

[14] Jones B.M., Rao V.D., Gernat T., Jagla T., Cash-Ahmed A.C., Rubin B.E., Comi T.J., Bhogale S., Husain S.S., Blatti C. (2020). Individual differences in honey bee behavior enabled by plasticity in brain gene regulatory networks. eLife.

[15] Arenas A., Ramírez G.P., Balbuena M.S., Farina W.M. (2013). Behavioral and neural plasticity caused by early social experiences: The case of the honeybee. Front. Physiol..

[16] Scheiner R., Baumann A., Blenau W. (2006). Aminergic control and modulation of honeybee behaviour. Curr. Neuropharmacol..

[17] Barbero F., Casacci L.P. (2025). The effect of biogenic amines in the neuromodulation of insect social behavior. Curr. Opin. Insect Sci..

[18] Farooqui T. (2007). Octopamine-mediated neuromodulation of insect senses. Neurochem. Res..

[19] Akülkü İ., Ghanem S., Filiztekin E., Suwannapong G., Mayack C. (2021). Age-Dependent Honey Bee Appetite Regulation Is Mediated by Trehalose and Octopamine Baseline Levels. Insects.

[20] Schulz D.J., Barron A.B., Robinson G.E. (2002). A role for octopamine in honey bee division of labor. Brain Behav. Evol..

[21] Barron A.B., Maleszka R., Vander Meer R.K., Robinson G.E. (2007). Octopamine modulates honey bee dance behavior. Proc. Natl. Acad. Sci. USA.

[22] Linn M., Glaser S.M., Peng T., Grüter C. (2020). Octopamine and dopamine mediate waggle dance following and information use in honeybees. Proc. Biol. Sci..

[23] Yellman C., Tao H., He B., Hirsh J. (1997). Conserved and sexually dimorphic behavioral responses to biogenic amines in decapitated *Drosophila*. Proc. Natl. Acad. Sci. USA.

[24] Maliszewska J., Jankowska M., Rogalska J. (2024). Octopamine is involved in TRP-induced thermopreference responses in *American cockroach*. J. Insect Physiol..

[25] Nakagawa H., Maehara S., Kume K., Ohta H., Tomita J. (2022). Biological functions of α2-adrenergic-like octopamine receptor in *Drosophila melanogaster*. Genes Brain Behav..

[26] Ma Z., Stork T., Bergles D.E., Freeman M.R. (2016). Neuromodulators signal through astrocytes to alter neural circuit activity and behaviour. Nature.

[27] Hu J., Bi R., Luo Y., Wu K., Jin S., Liu Z., Jia Y., Mao C.X. (2025). The gut microbiome promotes locomotion of *Drosophila* larvae via octopamine signaling. Insect Sci..

[28] Portugal R., Rodrigues B., Leitão R.A., Silva M., Pinheiro P.S., Carvalho A.L. (2025). Shaping the synapse through neuronal activity-regulated miRNAs. Trends Neurosci..

[29] Bitetti A., Mallory A.C., Golini E., Carrieri C., Carreño Gutiérrez H., Perlas E., Pérez-Rico Y.A., Tocchini-Valentini G.P., Enright A.J., Norton W.H.J. (2018). MicroRNA degradation by a conserved target RNA regulates animal behavior. Nat. Struct. Mol. Biol..

[30] Greenberg J.K., Xia J., Zhou X., Thatcher S.R., Gu X., Ament S.A., Newman T.C., Green P.J., Zhang W., Robinson G.E. (2012). Behavioral plasticity in honey bees is associated with differences in brain microRNA transcriptome. Genes Brain Behav..

[31] Jouravleva K., Zamore P.D. (2025). A guide to the biogenesis and functions of endogenous small non-coding RNAs in animals. Nat. Rev. Mol. Cell Biol..

[32] Martinetz S. (2016). MicroRNA’s impact on neurotransmitter and neuropeptide systems: Small but mighty mediators of anxiety. Pflug. Arch..

[33] Chen C.Y., Wang Y.F., Lei L., Zhang Y. (2025). MicroRNA-specific targets for neuronal plasticity, neurotransmitters, neurotrophic factors, and gut microbes in the pathogenesis and therapeutics of depression. Prog. Neuropsychopharmacol. Biol. Psychiatry.

[34] Sonawane S., Všianský V., Brázdil M. (2024). MicroRNA-mediated regulation of neurotransmitter receptors in epilepsy: A systematic review. Epilepsy Behav..

[35] Pulcrano S., De Gregorio R., De Sanctis C., Volpicelli F., Piscitelli R.M., Speranza L., Perrone-Capano C., di Porzio U., Caiazzo M., Martini A. (2023). miR-218 Promotes Dopaminergic Differentiation and Controls Neuron Excitability and Neurotransmitter Release through the Regulation of a Synaptic-Related Genes Network. J. Neurosci..

[36] Donelson N.C., Dixit R., Pichardo-Casas I., Chiu E.Y., Ohman R.T., Slawson J.B., Klein M., Fulga T.A., Van Vactor D., Griffith L.C. (2020). MicroRNAs Regulate Multiple Aspects of Locomotor Behavior in *Drosophila*. G3 Genes Genomes Genet..

[37] Issa A.R., Picao-Osorio J., Rito N., Chiappe M.E., Alonso C.R. (2019). A Single MicroRNA-Hox Gene Module Controls Equivalent Movements in Biomechanically Distinct Forms of *Drosophila*. Curr. Biol..

[38] Yang M., Du B., Xu L., Wang H., Wang Y., Lin K., He G., Kang L. (2023). Glutamate-GABA imbalance mediated by miR-8-5p and its STTM regulates phase-related behavior of locusts. Proc. Natl. Acad. Sci. USA.

[39] Yang M., Wei Y., Jiang F., Wang Y., Guo X., He J., Kang L. (2014). MicroRNA-133 inhibits behavioral aggregation by controlling dopamine synthesis in locusts. PLoS Genet..

[40] Liu F., Shi T., Yin W., Su X., Qi L., Huang Z.Y., Zhang S., Yu L. (2017). The microRNA ame-miR-279a regulates sucrose responsiveness of forager honey bees (*Apis mellifera*). Insect Biochem. Mol. Biol..

[41] Shi T., Zhu Y., Liu P., Ye L., Jiang X., Cao H., Yu L. (2021). Age and Behavior-Dependent Differential miRNAs Expression in the Hypopharyngeal Glands of Honeybees (*Apis mellifera* L.). Insects.

[42] Norain Sajid Z., Aziz M.A., Bodlah I., Rana R.M., Ghramh H.A., Khan K.A. (2020). Efficacy assessment of soft and hard acaricides against *Varroa destructor* mite infesting honey bee (*Apis mellifera*) colonies, through sugar roll method. Saudi J. Biol. Sci..

[43] Hamiduzzaman M.M., Emsen B., Hunt G.J., Subramanyam S., Williams C.E., Tsuruda J.M., Guzman-Novoa E. (2017). Differential Gene Expression Associated with Honey Bee Grooming Behavior in Response to *Varroa* Mites. Behav. Genet..

[44] Robinson M.D., McCarthy D.J., Smyth G.K. (2010). edgeR: A Bioconductor package for differential expression analysis of digital gene expression data. Bioinformatics.

[45] Huang J., Zhang Z., Feng W., Zhao Y., Aldanondo A., de Brito Sanchez M.G., Paoli M., Rolland A., Li Z., Nie H. (2022). Food wanting is mediated by transient activation of dopaminergic signaling in the honey bee brain. Science.

[46] Guo X., Ma Z., Du B., Li T., Li W., Xu L., He J., Kang L. (2018). Dop1 enhances conspecific olfactory attraction by inhibiting miR-9a maturation in locusts. Nat. Commun..

[47] Yang M., Wang Y., Jiang F., Song T., Wang H., Liu Q., Zhang J., Zhang J., Kang L. (2016). miR-71 and miR-263 Jointly Regulate Target Genes *Chitin synthase* and *Chitinase* to Control Locust Molting. PLoS Genet..

[48] Raza M.F., Li W. (2025). Biogenic amines in honey bee cognition: Neurochemical pathways and stress impacts. Curr. Opin. Insect Sci..

[49] Zhao W., Li Q., Cui F. (2020). Potential functional pathways of plant RNA virus-derived small RNAs in a vector insect. Methods.

[50] Groh C., Rössler W. (2020). Analysis of Synaptic Microcircuits in the Mushroom Bodies of the Honeybee. Insects.

[51] Caron S., Abbott L.F. (2017). Neuroscience: Intelligence in the Honeybee Mushroom Body. Curr. Biol..

[52] He J., Kang L. (2024). Regulation of insect behavior by non-coding RNAs. Sci. China Life Sci..

[53] Lucas K.J., Zhao B., Liu S., Raikhel A.S. (2015). Regulation of physiological processes by microRNAs in insects. Curr. Opin. Insect Sci..

[54] Pang X.D., Li Y.S., Lu R.H., Smagghe G., Liu T.X., Gui S.H. (2025). miR-7977 regulates the locomotor behavior by targeting diuretic hormone and SIFamide receptors in *Tribolium castaneum*. Int. J. Biol. Macromol..

[55] Dikmen F., Dabak T., Özgişi B.D., Özenirler Ç., Kuralay S.C., Çay S.B., Çınar Y.U., Obut O., Balcı M.A., Akbaba P. (2024). Transcriptome-wide analysis uncovers regulatory elements of the antennal transcriptome repertoire of bumblebee at different life stages. Insect. Mol. Biol..

[56] Ma Y.C., Zhang L., Dai L.L., Khan R.U., Zou C.G. (2017). mir-67 regulates *P. aeruginosa* avoidance behavior in *C. elegans*. Biochem. Biophys. Res. Commun..

[57] Horie T., Nakao T., Miyasaka Y., Nishino T., Matsumura S., Nakazeki F., Ide Y., Kimura M., Tsuji S., Rodriguez R.R. (2021). microRNA-33 maintains adaptive thermogenesis via enhanced sympathetic nerve activity. Nat. Commun..

[58] Huang M., Dong J., Guo H., Wang D. (2021). Effects of Dinotefuran on Brain miRNA Expression Profiles in Young Adult Honey Bees (*Hymenopptera*: *Apidae*). J. Insect Sci..

[59] Tianle C., Liuxu Y., Delong L., Yunhan F., Yu H., Xueqing S., Haitao X., Guizhi W. (2022). Fluvalinate-Induced Changes in MicroRNA Expression Profile of *Apis mellifera ligustica* Brain Tissue. Front. Genet..

[60] Depintor T.S., Freitas F.C.P., Hernandes N., Nunes F.M.F., Simões Z.L.P. (2025). Interactions of juvenile hormone, 20-hydroxyecdysone, developmental genes, and miRNAs during pupal development in *Apis mellifera*. Sci. Rep..

[61] Leinwand S.G., Scott K. (2021). Juvenile hormone drives the maturation of spontaneous mushroom body neural activity and learned behavior. Neuron.

[62] Luo J., Yi G., Liu S., Mei Y., Chen W., Hou J., Zhang F., Yang T., Li H., Li X. (2022). Juvenile Hormone III R Stereoisomer Is Specifically Synthesized by Honeybees (*Apis mellifera ligustica*) and Shows a Higher Biological Activity in Regulating Their Social Behavior. J. Agric. Food Chem..

[63] Giray T., Giovanetti M., West-Eberhard M.J. (2005). Juvenile hormone, reproduction, and worker behavior in the neotropical social wasp *Polistes canadensis*. Proc. Natl. Acad. Sci. USA.

[64] Guo S., Dong Y., Shu Y., Wu X., Li C., Ni Y., Zhang H., Ma W. (2026). MicroRNA-669g impairs serotonin balance through TPH2 downregulation and induces behavioral deficits. Behav. Brain Res..

[65] Shi S., Leites C., He D., Schwartz D., Moy W., Shi J., Duan J. (2014). MicroRNA-9 and microRNA-326 regulate human dopamine D2 receptor expression, and the microRNA-mediated expression regulation is altered by a genetic variant. J. Biol. Chem..

[66] Roeder T. (2005). Tyramine and octopamine: Ruling behavior and metabolism. Annu. Rev. Entomol..

[67] Kuo H.W. (2023). Tyramine beta hydroxylase-mediated octopamine synthesis pathway in *Litopenaeus vannamei* under thermal, salinity, and *Vibrio alginolyticus* infection stress. Fish Shellfish Immunol..

[68] Xu L., Jiang H.B., Chen X.F., Xiong Y., Lu X.P., Pei Y.X., Smagghe G., Wang J.J. (2018). How Tyramine β-Hydroxylase Controls the Production of Octopamine, Modulating the Mobility of Beetles. Int. J. Mol. Sci..

[69] Kaku N.G., Jankauski M.A., Doyle B.F., Collins C.J., Flenniken M.L. (2025). Inapparent virus infections differentially affect honey bee flight. Sci. Adv..

[70] Kaya-Zeeb S., Engelmayer L., Straßburger M., Bayer J., Bähre H., Seifert R., Scherf-Clavel O., Thamm M. (2022). Octopamine drives honeybee thermogenesis. eLife.

[71] Stolz T., Diesner M., Neupert S., Hess M.E., Delgado-Betancourt E., Pflüger H.J., Schmidt J. (2019). Descending octopaminergic neurons modulate sensory-evoked activity of thoracic motor neurons in stick insects. J. Neurophysiol..

[72] Kreissl S., Eichmüller S., Bicker G., Rapus J., Eckert M. (1994). Octopamine-like immunoreactivity in the brain and subesophageal ganglion of the honeybee. J. Comp. Neurol..

