# Supplementary material for: MicroRNA-281-X Modulates Self-Grooming Behavior in Honeybees by Targeting Tyrosine Decarboxylase 2 in the Octopaminergic Pathway
Source: Insects. 2026 May 20;17(5):522. doi: 10.3390/insects17050522 (PMC13207757; doi:10.3390/insects17050522)
Supplement: Supplementary file 1 [file insects-17-00522-s001.zip › insects-4289492-supplementary.pdf]

Supplementary Information

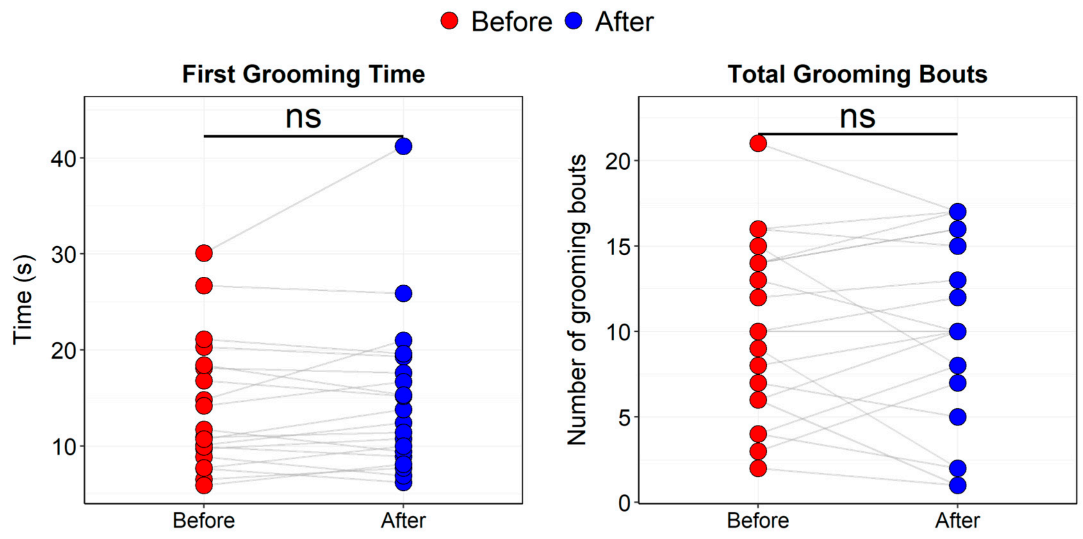

Figure S1. Neurotransmitters influence self-grooming behavior in *A. mellifera*. (A,B) MW bees received a topical application of DMF on the thorax as the control group. Time of the first grooming response and the total grooming bouts were quantified during 30–60 min periods (*n* = 20 per group, Wilcoxon signed-rank test).

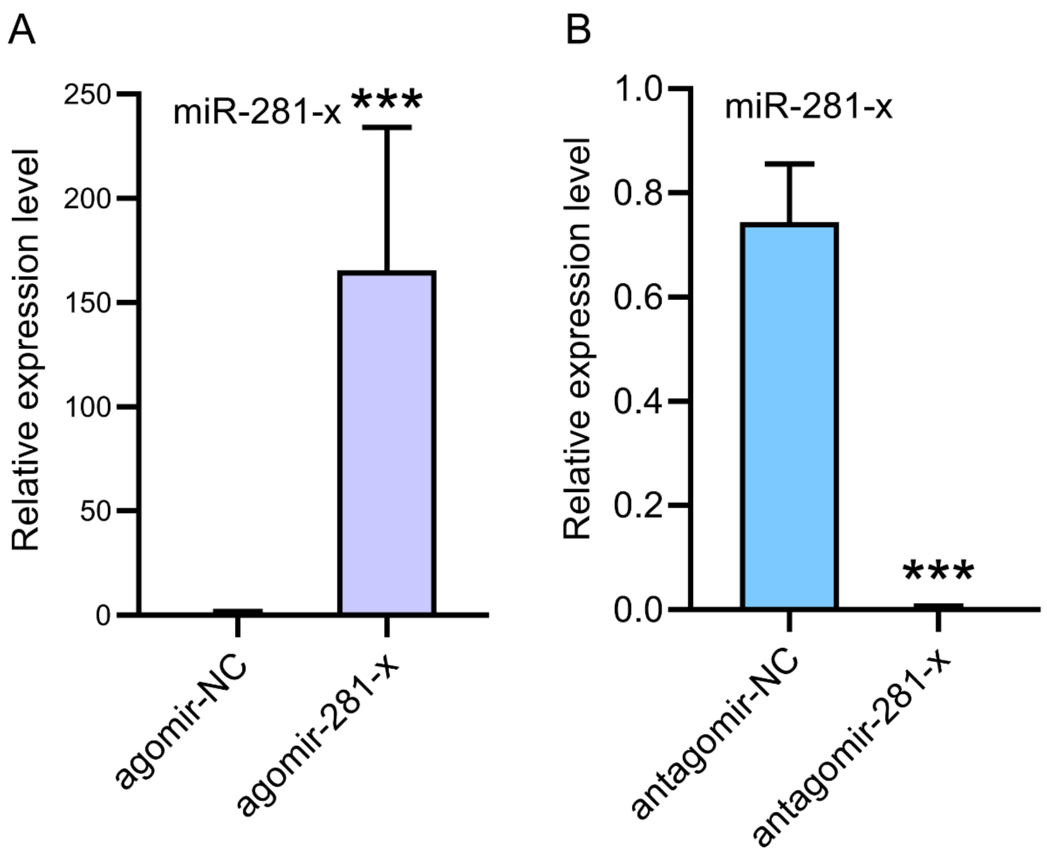

Figure S2. The expression level of miR-281-x of honeybees. (A,B) The miR-281-x relative expression was injected with agomir-281-x and antagomir-281-x (n = 5 per group). The data are presented as the mean ± SEM. The asterisks outside the strip indicate the significant difference between controls and the treatments by Student's t-test. *** $p < 0.001$.

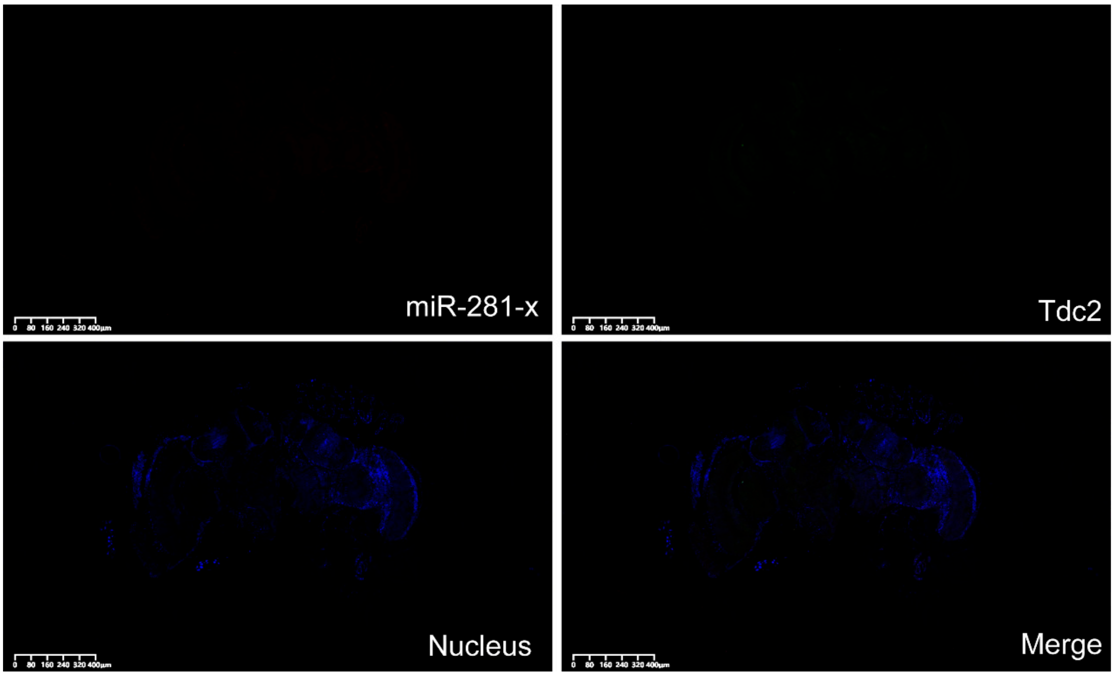

Figure S3. The negative control for co-localization of miR-281-x and *Tdc2* in the honeybee brain. The images were visualized using a confocal fluorescence microscope (Zeiss), Scran bar 400 μm.

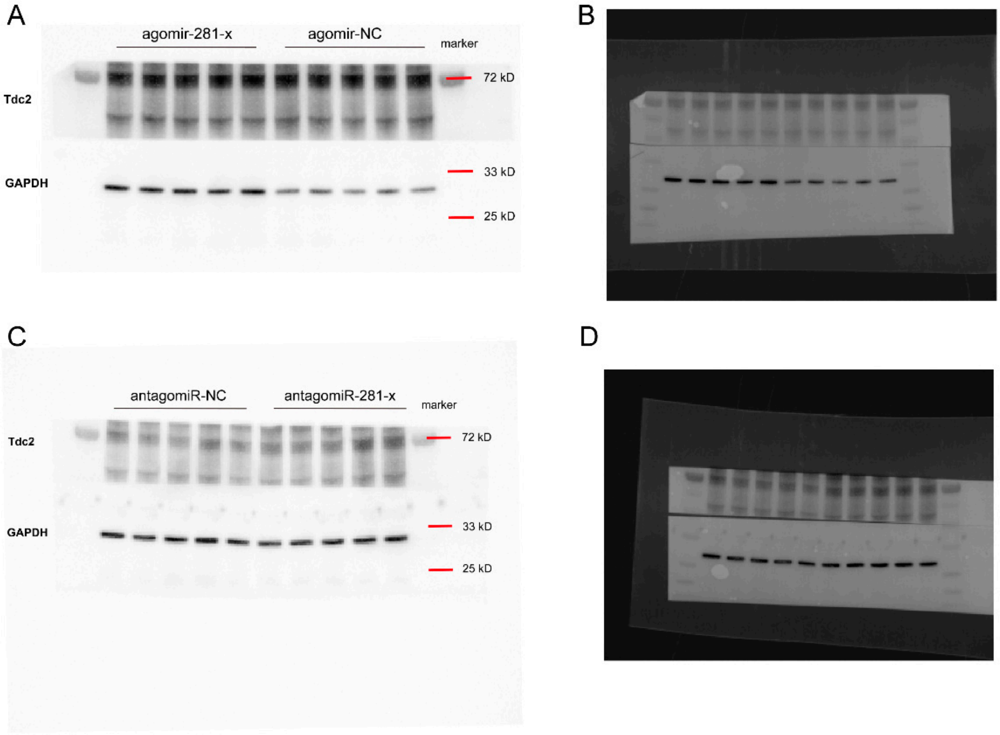

Figure S4. The western blot scans of Tdc2. (A,C) The western blot scans of Tdc2 in the honeybee brains injected with agomir-281-x and antagomir-281-x (*n* = 5 per group). (B,D) There were original images of agomir-281-x and antagomir-281-x, respectively.

A

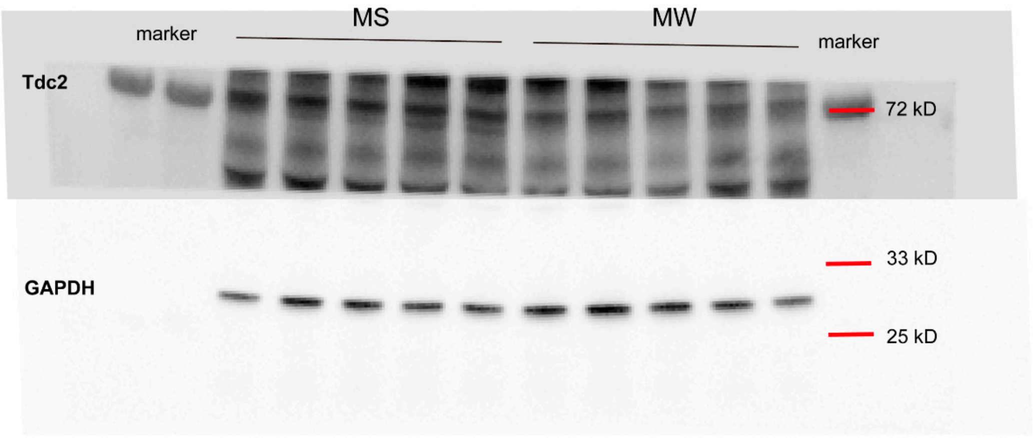

B

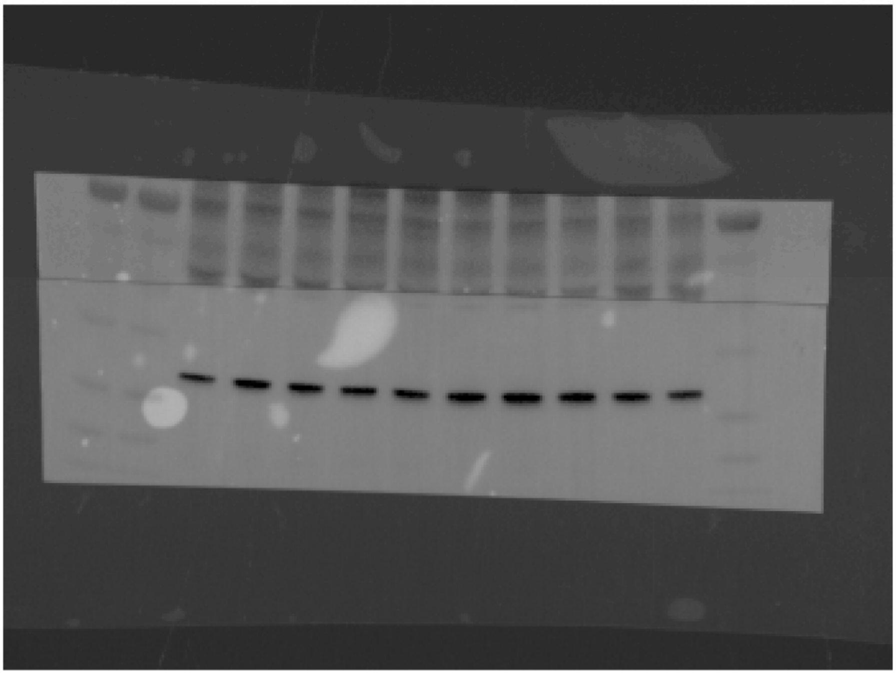

Figure S5. The western blot scans of Tdc2. (A,B) The western blot scan of Tdc2 in the MS and MW bee brains and the original image (n = 5 per group).

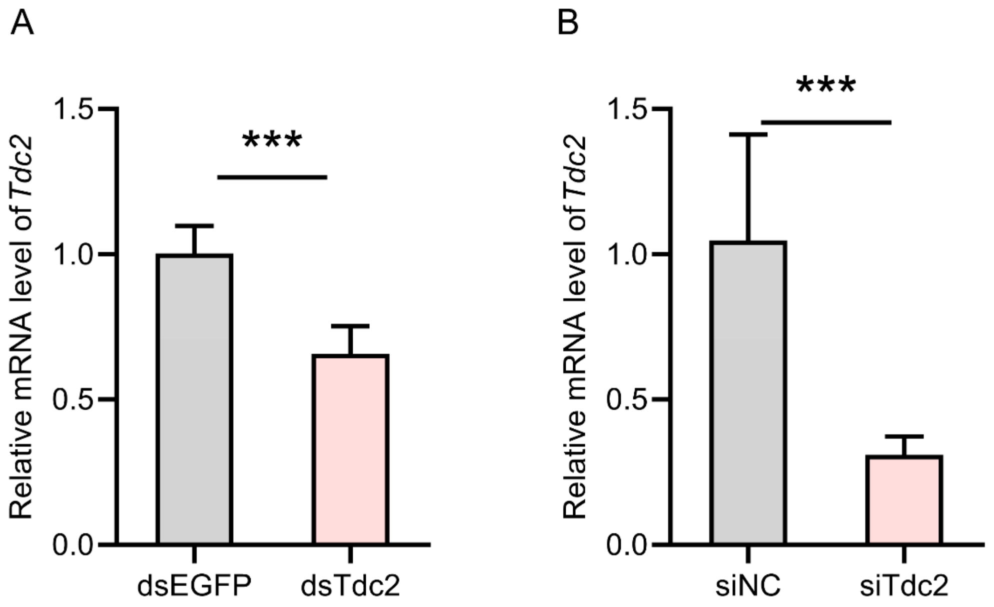

Figure S6. The expression level of *tdc2* of honeybees. (A,B) The *tdc2* relative expression was injected with dsEGFP and dstdc2 or siNC and sitdc2 (n = 6 per group). The data are presented as the mean ± SEM; The asterisks outside the strip indicate the significant difference between controls and the treatments by Student's t-test. *** $p < 0.001$.

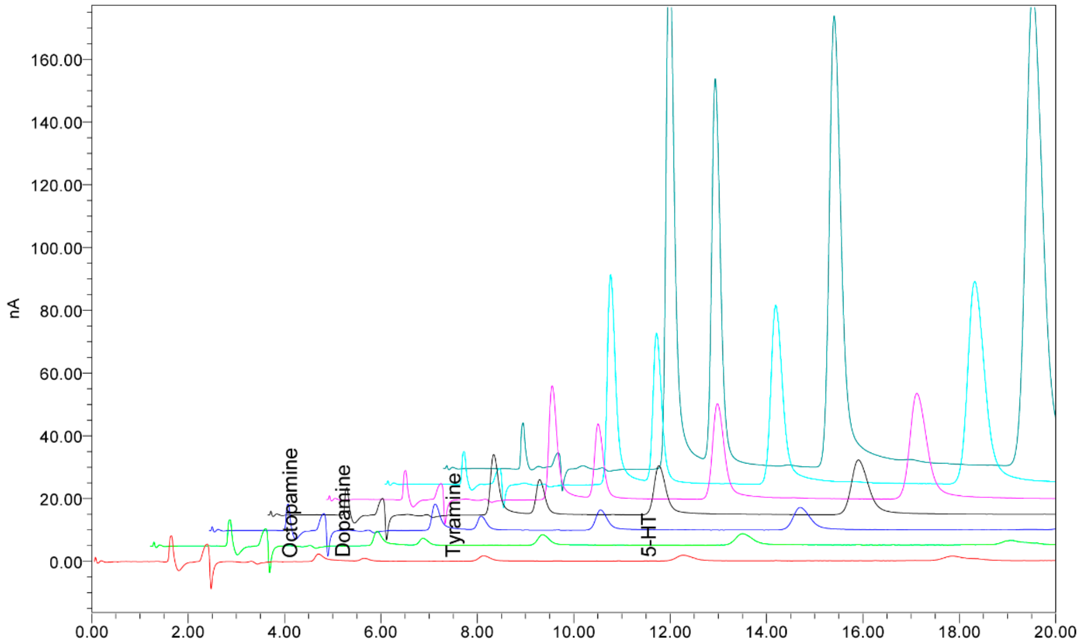

Figure S7. HPLC-ECD chromatogram of biogenic amines. Depicts a representative calibration curve using octopamine, dopamine, tyramine, and serotonin standards ranging from 5 ng to 0.5 µg. Red line: 5ng/mL; green line: 10 ng/mL; black line: 20 ng/mL; purple line: 50 ng/mL; pink line: 100 ng/mL; blue line: 200 ng/mL; cyan line: 500 ng/mL.

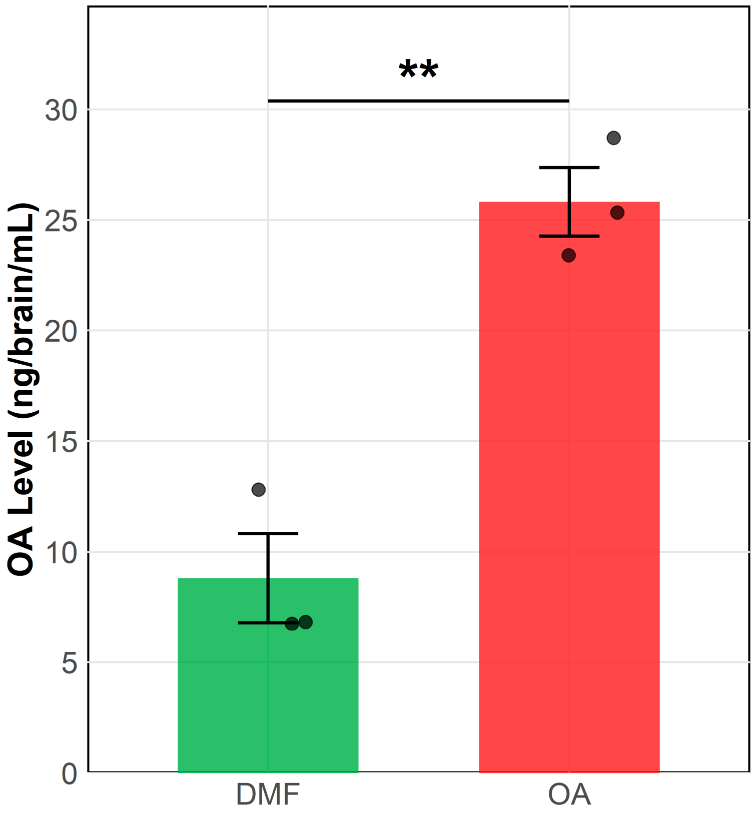

Figure S8. Octopamine levels in honeybee brain 30 min after topical application of DMF or 5 μg OA. Data are shown as mean ± SEM. ** $p < 0.01$, unpaired two-tailed Student's *t*-test ($n = 3$ per group).

Supplementary Table S1. Primers used in this study.

Gene/miRNA	Sequence (5' to 3')	Experiments
miR-281-x	GTCGTATCCAGTGCAGGGTCCGAGGTATTCGCACTGGATACGACCTGTCG	miRNA cDNA Synthesis
ame-miR-1-3p	GTCGTATCCAGTGCAGGGTCCGAGGTATTCGCACTGGATACGACCTCCTC	
ame-miR-12-5p	GTCGTATCCAGTGCAGGGTCCGAGGTATTCGCACTGGATACGACCCGTCC	
ame-miR-10-5p	GTCGTATCCAGTGCAGGGTCCGAGGTATTCGCACTGGATACGACCTTCGG	
ame-miR-375-3p	GTCGTATCCAGTGCAGGGTCCGAGGTATTCGCACTGGATACGACTAACTC	
ame-miR-9a-5p	GTCGTATCCAGTGCAGGGTCCGAGGTATTCGCACTGGATACGACTCTCGC	
ame-miR-263a-5p	GTCGTATCCAGTGCAGGGTCCGAGGTATTCGCACTGGATACGACGTGTTC	
U6	TATCAGGCATTTCTCCACCAG	
miR-281-x-F	GCGCGAAGAGAGCACCA	qPCR
ame-miR-1-3p-F	CGCGCGGGAAGAAAGAA	
ame-miR-12-5p-F	GCGCGCGGAGAACACA	
ame-miR-10-5p-F	CGCGCGCGACCCGAGA	
ame-miR-375-3p-F	GCGTTTGTTCGTTCGGCTC	
ame-miR-9a-5p-F	CGCGCGCGCGCGGACA	
ame-miR-263a-5p-F	GCGGAAAGGCACGGAA	
Revise miR primer	AGTGCAGGGTCCGAGGTATT	
U6-F	CGGAAGGAAGAAGAGTCACCA	
U6-R	TATCAGGCATTTCTCCACCAG	
tdc2-F	CCGAGCCTTGGGAGAACATT	
tdc2-R	TTTCCCGAACCAATCGCAGA	
Actin-F	TTGTATGCCAACACTGTCCTTT	
Actin-R	TGGCGCGATGATCTTAATTT	
dstdc2-F	CGTTGGTCGTCGATCCTCTT	dsRNA/siRNA synthesis
dstdc2-R	CAGCATCTTTCGCCTGCATC	
T7-*dstdc2*-F	TAATACGACTCACTATAGGGCGTTGGTCGTCGATCCT	

	CTT	
T7-*dstdc2*-R	TAATACGACTCACTATAGGGCAGCATCTTTCGCCTGCATC	
dsEGFP-F	ATGAGCAAGGGCGAGGAACTG	
dsEGFP-R	CTTGTACAGCTCGTCCATGCC	
T7-*dsEGFP*-F	GGATCCTAATACGACTCACTATAGGATGAGCAAGGGCGAGGAACTG	
T7-*dsEGFP*-R	GGATCCTAATACGACTCACTATAGGCTTGTACAGCTCGTCCATGCC	
sitdc2-F	GGCGAAAGAUGCUGCUGAU(dT)(dT)	
sitdc2-R	AUCAGCAGCAUCUUUCGCC(dT)(dT)	
pmirGLO-F	CGAGGTGCCTAAAGGACTGACCG	Luciferase report assay
pmirGLO-R	ACTGCATTCTAGTTGTGGTTTGTCC	
Mutation-F	AGTCTcgagagaTTGGATCGCGACAGAGATGC	
Mutation-R	TCCAAtctctcgAGACTTATATAAACAGATCGATAAGCAAGTA	
agomir-NC	UUCUCCGAACGUGUCACGUTT<br>ACGUGACACGUUCGGAGAATT	miRNA mimic/ inhibitor synthesis
antagomir NC	CAGUACUUUUGUGUAGUACAA	
miR-281-x-agomir	AAGAGAGCUAUCCAUCGACAGU<br>UGUCGAUGGAUAGCUCUCUUUU	
miR-281-x-antagomir	ACUGUCGAUGGAUAGCUCUCUU	

Supplementary Table S2. Probe sequence used in this study.

Gene/miRNA	Sequence (5' to 3')	Experiments
DIG-miR-281-x	ACTGTCGATGGATAGCTCTCTT	FISH
DIG-*Tdc2*	GAATCGATGGAAAGGAATTTCCGGCTGG	
	CAACAAGCAGTCGTGCCTAAAGTGGT	
	TACCAGCCATCAGGTACTTCAATTCCGG	
	CAAGCGCGAAACGAATAGTGAACCGT	
	GCGACAATTCACTGGTATCAGCAGCATC	
